# Development of ^68^Ga-labeled macrocyclic peptide for targeting stimulator of interferon genes

**DOI:** 10.1016/j.apsb.2026.03.010

**Published:** 2026-03-11

**Authors:** Jingru Zhang, Lingxin Meng, Huifeng Li, Dongsheng Xia, Xiaobo Wang, Rongqiang Zhuang, Huanhuan Liu, Yesen Li, Xianzhong Zhang, Jianyang Fang, Zhide Guo

**Affiliations:** a State Key Laboratory of Vaccines for Infectious Diseases, Xiang An Biomedicine Laboratory, National Innovation Platform for Industry-Education Integration in Vaccine Research, Center for Molecular Imaging and Translational Medicine, School of Public Health, Xiamen University, Xiamen 361102, China; b School of Medical Imaging, Fujian Medical University, Fuzhou 350122, China; c Department of Nuclear Medicine, The First Affiliated Hospital of Xiamen University, School of Medicine, Xiamen University, Xiamen 361003, China; d Zhangzhou Affiliated Hospital of Fujian Medical University, Zhangzhou 363000, China; e Theranostics and Translational Research Center, National Infrastructures for Translational Medicine, Institute of Clinical Medicine & Department of Nuclear Medicine, Peking Union Medical College Hospital, Chinese Academy of Medical Sciences & Peking Union Medical College, Beijing 100730, China; f Department of Nuclear Medicine and State Key Laboratory of Holistic Integrative Management of Gastrointestinal Cancers, Xijing Hospital, Fourth Military Medical University, Xi’an 710032, China

**Keywords:** STING, Macrocyclic peptide, Gallium-68, Tumor microenvironment, PET imaging, Immunotherapy, Radiotracer, Doxorubicin

## Abstract

The stimulator of interferon genes (STING) plays a vital role in innate immunity and is a promising anticancer target. Visualizing STING expression is essential for optimizing and guiding STING-targeted immunotherapies. This study aimed to design and develop macrocyclic peptide radiotracers for evaluating STING expression within the tumor microenvironment. A series of macrocyclic peptide-targeted STING radiotracers ([^68^Ga]Ga-FMAD, [^68^Ga]Ga-FMTD, [^68^Ga]Ga-FMT2D, and [^68^Ga]Ga-FMT3D) were synthesized by modifying the amino acid sequences and linkers. The radiotracers exhibited high radiochemical yield, radiochemical purity, and molar activity. PET imaging of [^68^Ga]Ga-FMAD and [^68^Ga]Ga-FMTD showed suboptimal tumor uptake, indicating limited STING-targeting efficacy. [^68^Ga]Ga-FMT3D demonstrated tumor uptake comparable to that of [^68^Ga]Ga-FMT2D while exhibiting superior pharmacokinetic properties, as indicated by a higher tumor-to-muscle (T/M) ratio. The tumor uptake values of [^68^Ga]Ga-FMT2D and [^68^Ga]Ga-FMT3D significantly decreased after NNK-2 (STING-specific binding compound) preadministration, indicating STING-specific targeting. The competition assays demonstrated high affinities, with IC_50_ values of 8.81 and 5.25 nmol/L for [^68^Ga]Ga-FMT2D and [^68^Ga]Ga-FMT3D, respectively. Furthermore, tumor uptake of [^68^Ga]Ga-FMT3D was significantly correlated with STING protein levels in the doxorubicin-treated model. Consequently, [^68^Ga]Ga-FMT3D, exhibiting high affinity for and specificity to STING, possesses favorable pharmacokinetics, serving as a promising tool for quantifying STING expression within the tumor microenvironment.

## Graphical abstract

This study reported rational design of ^68^Ga-labeled macrocyclic peptide for STING-targeted imaging in tumor microenvironment.

**Figure:**
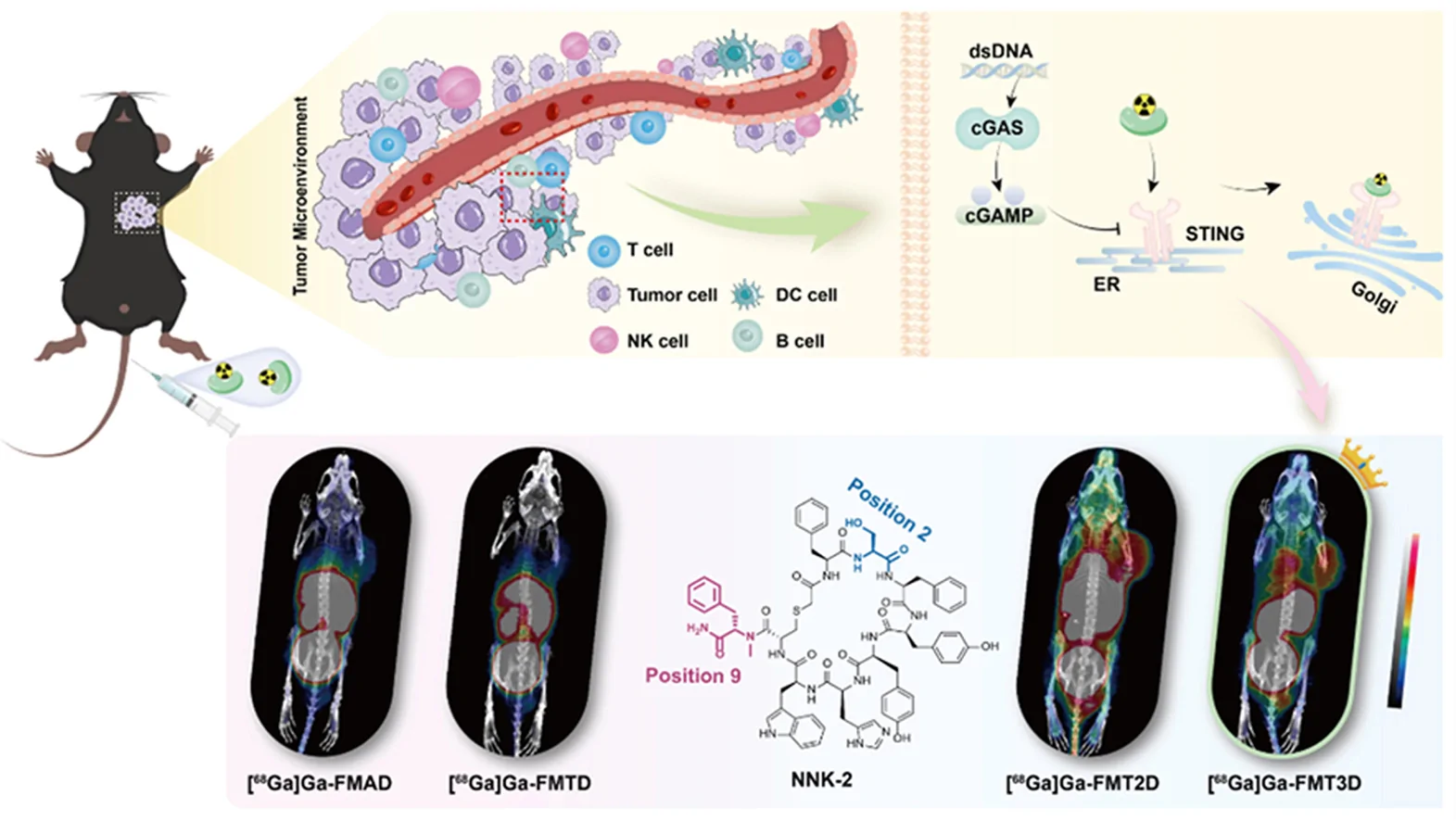

## 1 Introduction

The cyclic GMP-AMP synthase (cGAS) - stimulator of interferon genes (STING) pathway promote cancer immunity^1^, ^2^, ^3^, ^4^. STING activation can trigger a downstream event that induces the production of type I interferon (IFN-I)^5^, ^6^, ^7^. IFN-I can elicit antigen-specific adaptive immune responses by fostering the maturation, differentiation, and mobilization of immune effectors in the tumor microenvironment. This subsequently increases the response rate to existing cancer treatments such as radiotherapies, chemotherapies, and immune therapy^8^, ^9^, ^10^, ^11^. Consequently, improvements in small-molecule agonists targeting STING as a novel category of immuno-oncology agents have been reported^12^, ^13^, ^14^, ^15^, ^16^. Given the widespread expression of STING protein in the body, patient screening should be performed with care to avoid “cytokine storm” in normal tissues. The results also indicated that STING could reflect the immune status in the tumor microenvironment: high STING levels in tumors were positively correlated with immune cell infiltration, whereas lower levels were associated with poor prognosis in patients with colorectal, gastric, and lung cancers^17^, ^18^, ^19^. In conclusion, STING is a valuable marker for clinical treatment decision-making, including patient management, efficacy monitoring, and prognostic prediction.

Immunohistochemistry (IHC) of biopsied specimens is limited by inter- and intralesion heterogeneities and cannot assess the amount of whole-tumor STING that may influence immunotherapy efficacy. Positron emission tomography (PET) is a highly sensitive, high-resolution imaging modality in the clinic and can overcome the limitations of IHC, such as sampling error, invasiveness, and difficult serial examinations^20^^,^^21^. Importantly, STING expression exhibits dynamic changes during disease progression and therapeutic intervention that are not readily captured by static IHC-based assessments. Developing radioligand targeting STING to assess STING expression levels in tumors using PET is necessary. Current molecular imaging agents targeting STING have primarily focused on the development of its small-molecule agonists and inhibitors. In our previous studies, well-known STING agonists, aminobenzimidazole (ABZI) and 5,6-dimethylxanthenone-4-acetic acid (DMXAA), were used to develop [^18^F]F-ABI, [^18^F]F-DABI, and [^18^F]F-CRI_*n*_, and demonstrated their feasibility in visualizing STING *in vivo*^22^, ^23^, ^24^, ^25^. In addition, radiolabeled STING inhibitors [^131^I]I-NFIP and [^18^F]F-NFEP were developed to validate their potential for evaluating STING expression in the PANC02 tumor model^26^. Furthermore, Xu et al. developed an ^18^F-labeled STING agonist tracer, [^18^F]FBTA, which was used to diagnose early acute lung injury or myocarditis and monitor the immunogenicity of colorectal cancer^27^^,^^28^. Collectively, these radiotracers have demonstrated the feasibility of noninvasive evaluation of STING expression. Nevertheless, challenges remain, as the above radiotracers exhibit off-target uptake in the gastrointestinal tract and gallbladder, which may reduce imaging contrast and hinder clinical translation. These limitations have motivated us to develop new STING-targeted radiotracers with better imaging contrast and favorable *in vivo* pharmacokinetic properties.

Peptide-based radiopharmaceuticals have gained significant acceptance in diagnostic imaging and targeted therapy^29^. Several notable examples, such as [^68^Ga]Ga-DOTATATE, Lutathera, and [^68^Ga]Ga-FAP-2286, have achieved clinical success^30^, ^31^, ^32^, ^33^, ^34^. Cyclic peptide-based radiotracers have potentially enhanced biological properties compared with their linear counterparts, such as greater binding affinity and selectivity due to their conformational rigidity and improved plasma stability^35^. Radioligand development based on cyclic peptides for targeting STING is a promising approach that contributes significantly to the STING visualization probe toolbox. However, to our knowledge, no peptide-based STING-targeted radiotracers have been reported.

In light of the current research state, this study have two objectives: (1) to develop and establish radiolabeled macrocyclic peptides for STING-targeted imaging in tumor-bearing mice and (2) explore optimization strategies to enhance the pharmacokinetic properties of the probes. Thus, in this study, the first STING-targeting macrocyclic peptide, NNK-2, which has a STING protein binding affinity of 3.4 nmol/L, was selected as the lead compound^36^. Rational amino acid replacement and linker regulation are well established for improving the pharmacokinetics of radiotracer. Through these structural optimization strategies, four radiotracers (denoted as [^68^Ga]Ga-FMAD, [^68^Ga]Ga-FMTD, [^68^Ga]Ga-FMT2D, and [^68^Ga]Ga-FMT3D) were designed, and their physicochemical properties, STING specificity and affinity, were explored (Fig. 1). In addition, the capacity of representative probes, [^68^Ga]Ga-FMT3D, to noninvasively evaluate STING expression in tumors was verified before and after doxorubicin (DOX) therapy.

**Figure 1:**
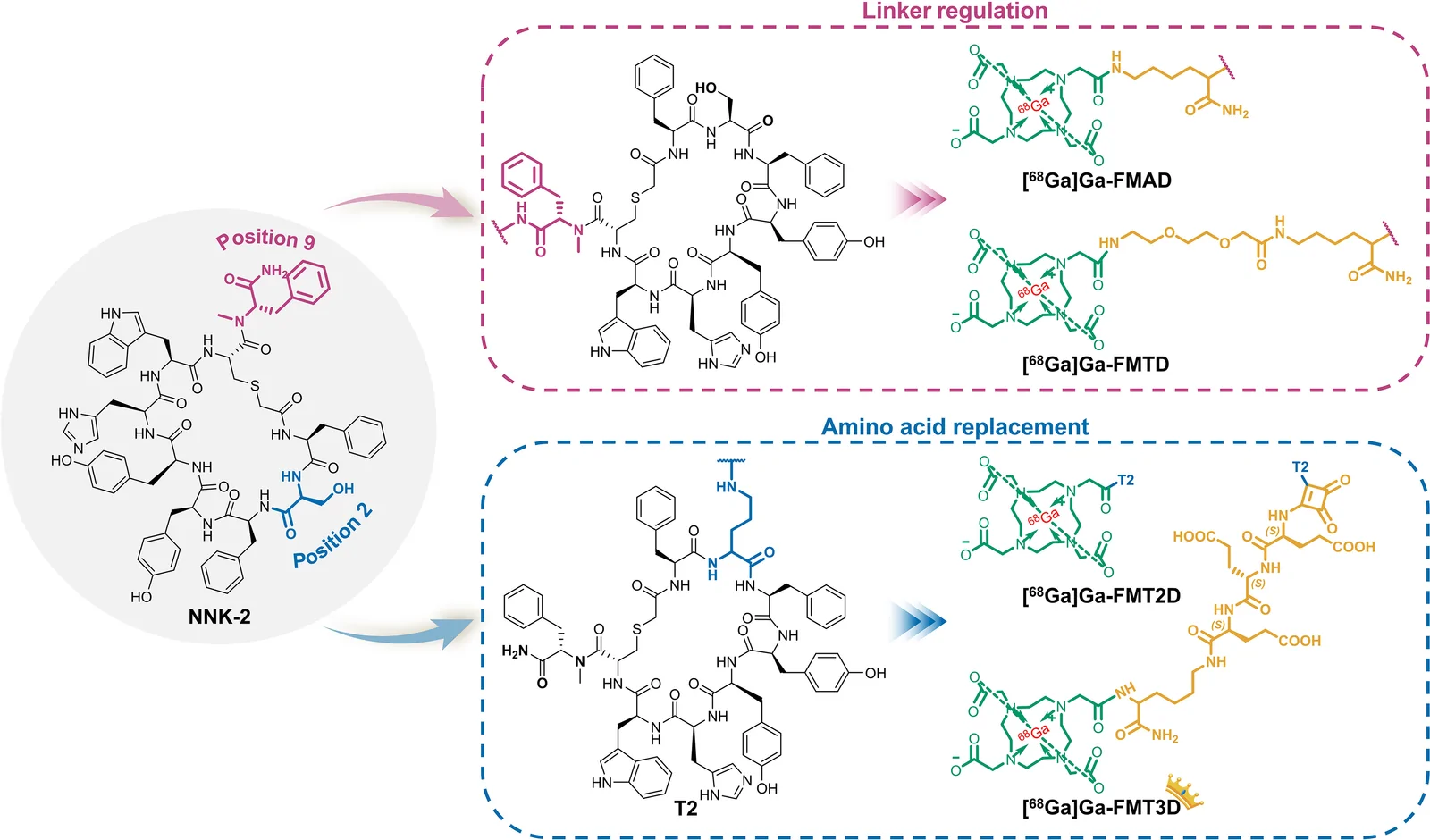
Chemical structures of the [^68^Ga]Ga-FMAD, [^68^Ga]Ga-FMTD, [^68^Ga]Ga-FMT2D, and [^68^Ga]Ga-FMT3D.

## 2 Results

### 2.1 Chemistry and radiochemistry

Fig. 1 and Supporting Information Fig. S9 shows the chemical structures of the [^68^Ga]Ga-FMAD, [^68^Ga]Ga-FMTD, [^68^Ga]Ga-FMT2D, and [^68^Ga]Ga-FMT3D. The detailed synthesis routes of precursors are shown in Supporting Information Figs. S1 and S2. The precursors were confirmed using ESI-MS (Supporting Information Figs. S4−S8), and chemical purity was determined by high-performance liquid chromatography (HPLC) with chemical purity >93% (Supporting Information Fig. S3).

The radiochemical yield (RCY), radiochemical purity (RCP), and molar activities of [^68^Ga]Ga-FMAD, [^68^Ga]Ga-FMTD, [^68^Ga]Ga-FMT2D, and [^68^Ga]Ga-FMT3D are summarized in Supporting Information Fig. S10, Table S1. The Log*P* values of [^68^Ga]Ga-FMAD, [^68^Ga]Ga-FMTD, [^68^Ga]Ga-FMT2D, and [^68^Ga]Ga-FMT3D were −1.55 ± 0.01, −2.12 ± 0.02, −0.55 ± 0.01, and −1.77 ± 0.02, respectively, presenting their hydrophilic properties.

### 2.2 Stability studies

According to the *in vitro* stability results shown in Supporting Information Figs. S11 and S12, [^68^Ga]Ga-FMAD, [^68^Ga]Ga-FMTD, [^68^Ga]Ga-FMT2D, and [^68^Ga]Ga-FMT3D exhibited excellent stability in both PBS and mouse serum. Following a 120 min coincubation period, no significant new radioactive peaks were detected, and the RCP of the radiotracers remained consistently above 90%. Moreover, the metabolic stability was evaluated in C57BL/6J male mice, in which [^68^Ga]Ga-FMT3D presented high stability in the urine and blood at 30 min p.i. (Supporting Information Fig. S13).

### 2.3 Molecular docking assays

Molecular docking analyses were performed to explore the interaction modes of NNK-2, ^nat^Ga-FMAD, ^nat^Ga-FMTD, ^nat^Ga-FMT2D, and ^nat^Ga-FMT3D with the STING protein (Fig. 2A–E). All ligands bound to the STING pocket and formed favorable polar interactions. Among them, NNK-2 formed hydrogen bonds with Arg238 and Ser241. ^nat^Ga-FMAD created hydrogen bonds with Val239, Gln266, and Asp210 and forms *π–π* stacking with Tyr167. ^nat^Ga-FMTD formed hydrogen bonds with Ser241, Leu170, and Gln228, whereas the Pi-Pi stacked with Tyr167. ^nat^Ga-FMT2D formed hydrogen bonds with Arg238 and Thr263. ^nat^Ga-FMT3D exhibited hydrogen bond interaction with Asp210 and Gln273. As shown in Fig. 2F, the docking scores of NNK-2, ^nat^Ga-FMAD, ^nat^Ga-FMTD, ^nat^Ga-FMT2D, and ^nat^Ga-FMT3D to the STING protein were measured as −13.21, −14.21, −14.83, −14.18, and −15.03 kcal/mol, respectively. Compared with other ligands, ^nat^Ga-FMT3D demonstrated a higher binding affinity to the STING protein.

**Figure 2:**
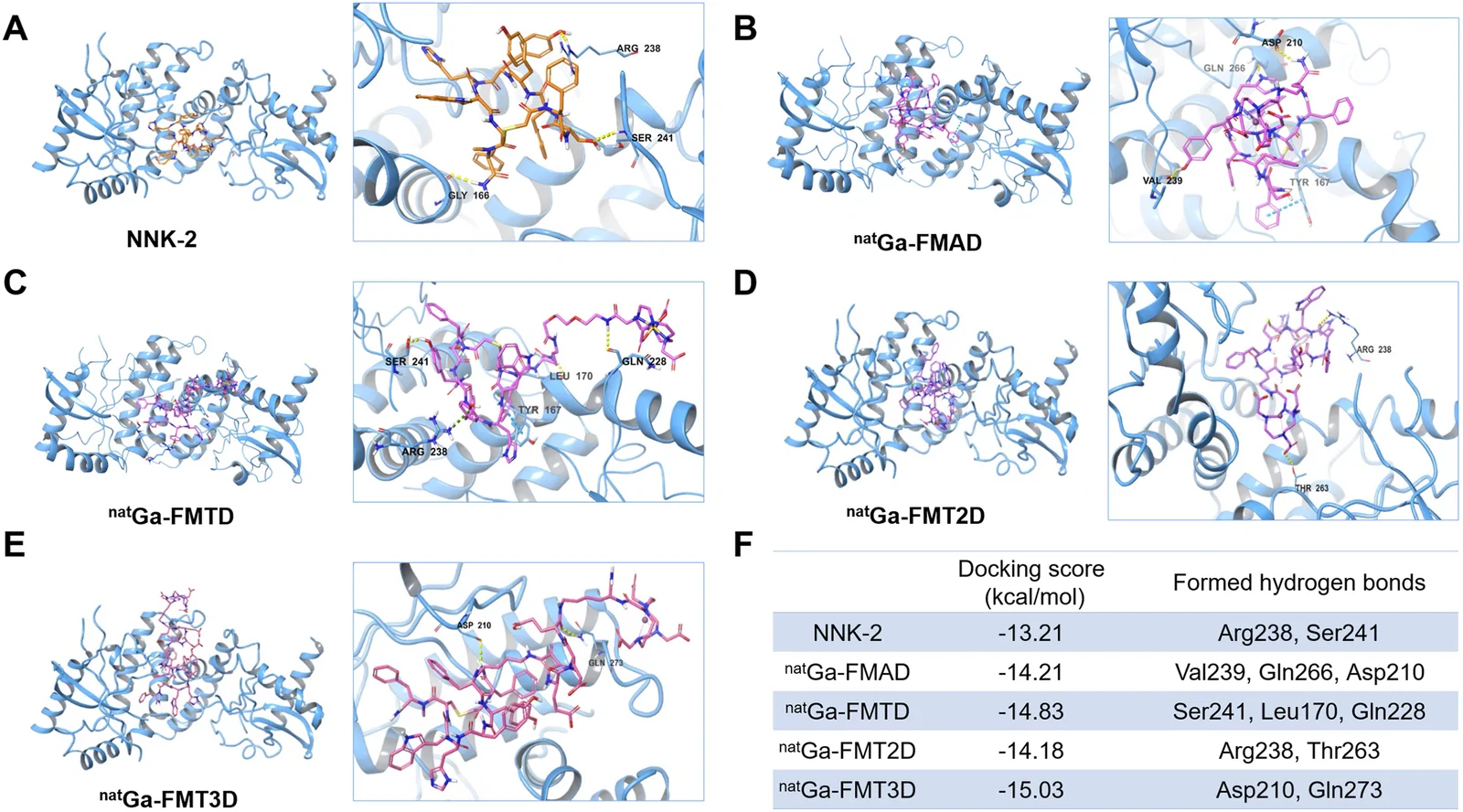
Molecular docking analyses. The binding modes of NNK-2 (A), ^nat^Ga-FMAD (B), ^nat^Ga-FMTD (C), ^nat^Ga-FMT2D (D), and ^nat^Ga-FMT3D (E) with the STING protein. (F) Docking score and formed hydrogen bonds of NNK-2, ^nat^Ga-FMAD, ^nat^Ga-FMTD, ^nat^Ga-FMT2D, and ^nat^Ga-FMT3D with the STING protein.

### 2.4 PET imaging

PET was performed to evaluate the *in vivo* STING-targeting ability of radiotracers in B16F10 tumor-bearing mice. As shown in Fig. 3A, all radiotracers accumulated in the tumors at 1 h p.i. In comparison to the tumor uptake of [^68^Ga]Ga-FMT2D (2.50 ± 0.10%ID/g), both [^68^Ga]Ga-FMAD (0.93 ± 0.05%ID/g) and [^68^Ga]Ga-FMTD (0.88 ± 0.05%ID/g) demonstrated significantly lower uptake values (∗∗∗∗*P* < 0.0001). In contrast, [^68^Ga]Ga-FMT3D showed comparable tumor uptake (2.3 ± 0.26%ID/g) with no statistically significant difference (*P* > 0.05). (Fig. 3B). The results indicate that modifications at position 9 of NNK-2 significantly affected the properties of the macrocyclic peptide. Notably, while [^68^Ga]Ga-FMT2D displayed prominent radioactivity accumulation in the abdominal region, [^68^Ga]Ga-FMT3D exhibited a markedly distinct biodistribution pattern. This differential biodistribution profile, likely attributable to structural modifications in the linker region. Specifically, the introduction of a glutamic acid moiety increases the polarity of the radioligand, shifts the lipophilicity-hydrophilicity balance, and promotes renal clearance, thereby reducing nonspecific abdominal uptake and improving tumor-to-muscle (T/M) ratio for [^68^Ga]Ga-FMT3D ([^68^Ga]Ga-FMT2D: 4.49 ± 0.18 *vs* [^68^Ga]Ga-FMT3D: 8.40 ± 0.04, ∗∗∗∗*P* < 0.0001) (Fig. 3C).

**Figure 3:**
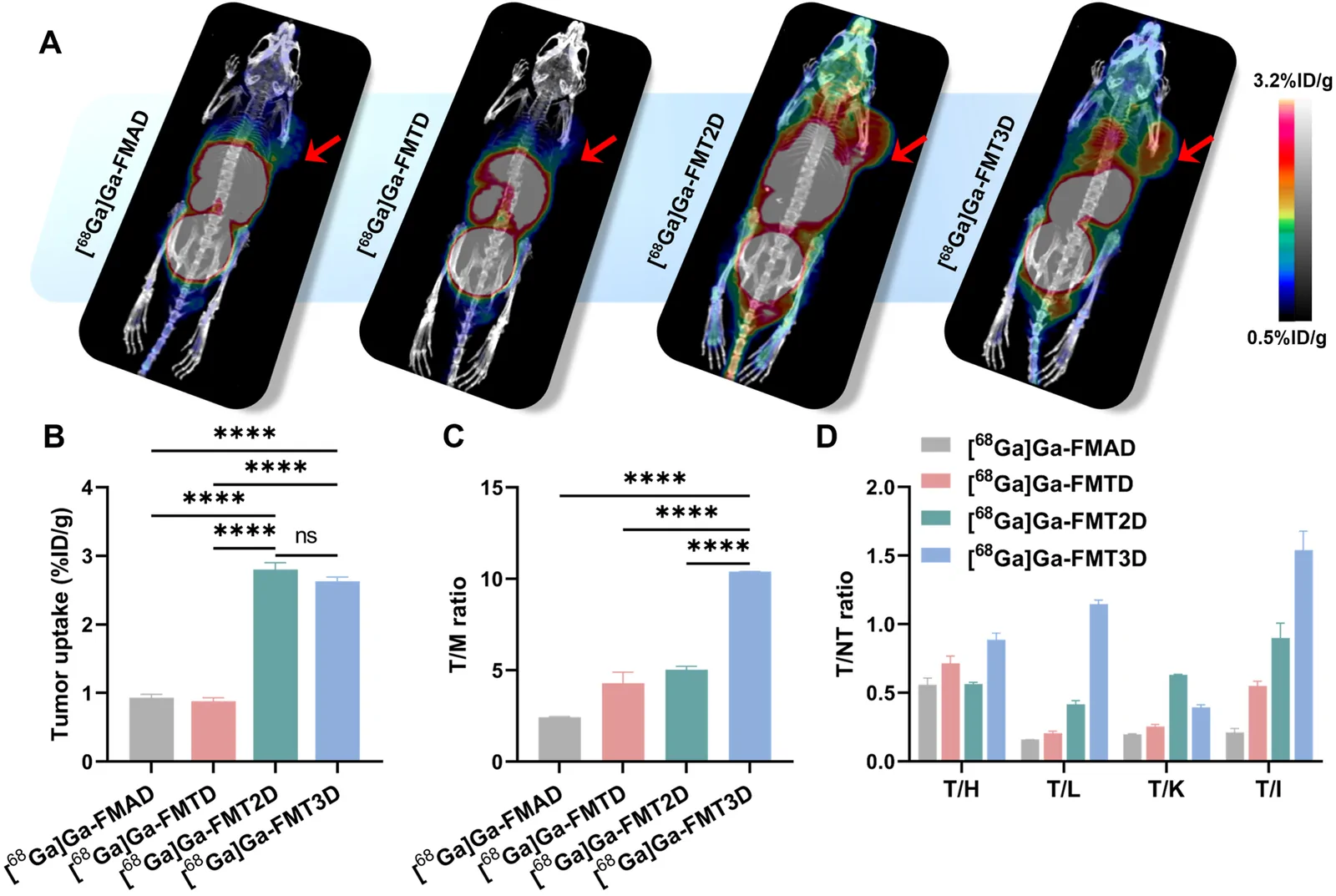
Discovery and validation of ^68^Ga-labeled macrocyclic peptides for STING imaging. (A) Representative maximum intensity projection (MIP) images of radiotracers in B16F10 tumor-bearing mice at 1 h p.i. The tumor uptake (B), tumor-to-muscle (T/M) ratio (C), and tumor-to-normal tissue (T/NT) ratio (D) of radiotracers were derived from PET images. T: tumor, M: muscle, H: heart (including contents), L: liver, K: kidney, I: intestine (^∗∗∗∗^*P* < 0.0001, ns: non significance).

### 2.5 *In vitro* studies

The cell uptake and competition assays of [^68^Ga]Ga-FMT2D and [^68^Ga]Ga-FMT3D were performed in Raw 264.7 cells, which exhibit high STING expression. In the cell uptake assays, [^68^Ga]Ga-FMT2D and [^68^Ga]Ga-FMT3D were coincubated with Raw 264.7 cells at 37 °C for 1 h. Compared with the normal group, the cellular uptake values of [^68^Ga]Ga-FMT2D and [^68^Ga]Ga-FMT3D in the blocking group pretreated with 10 μg NNK-2 were reduced significantly (∗∗*P* < 0.01). In the competition assays, the IC_50_ values of [^68^Ga]Ga-FMT2D and [^68^Ga]Ga-FMT3D were 8.81 and 5.25 nmol/L, respectively, indicating that the affinity of [^68^Ga]Ga-FMT3D is higher than that of [^68^Ga]Ga-FMT2D (Fig. 4A and B).

**Figure 4:**
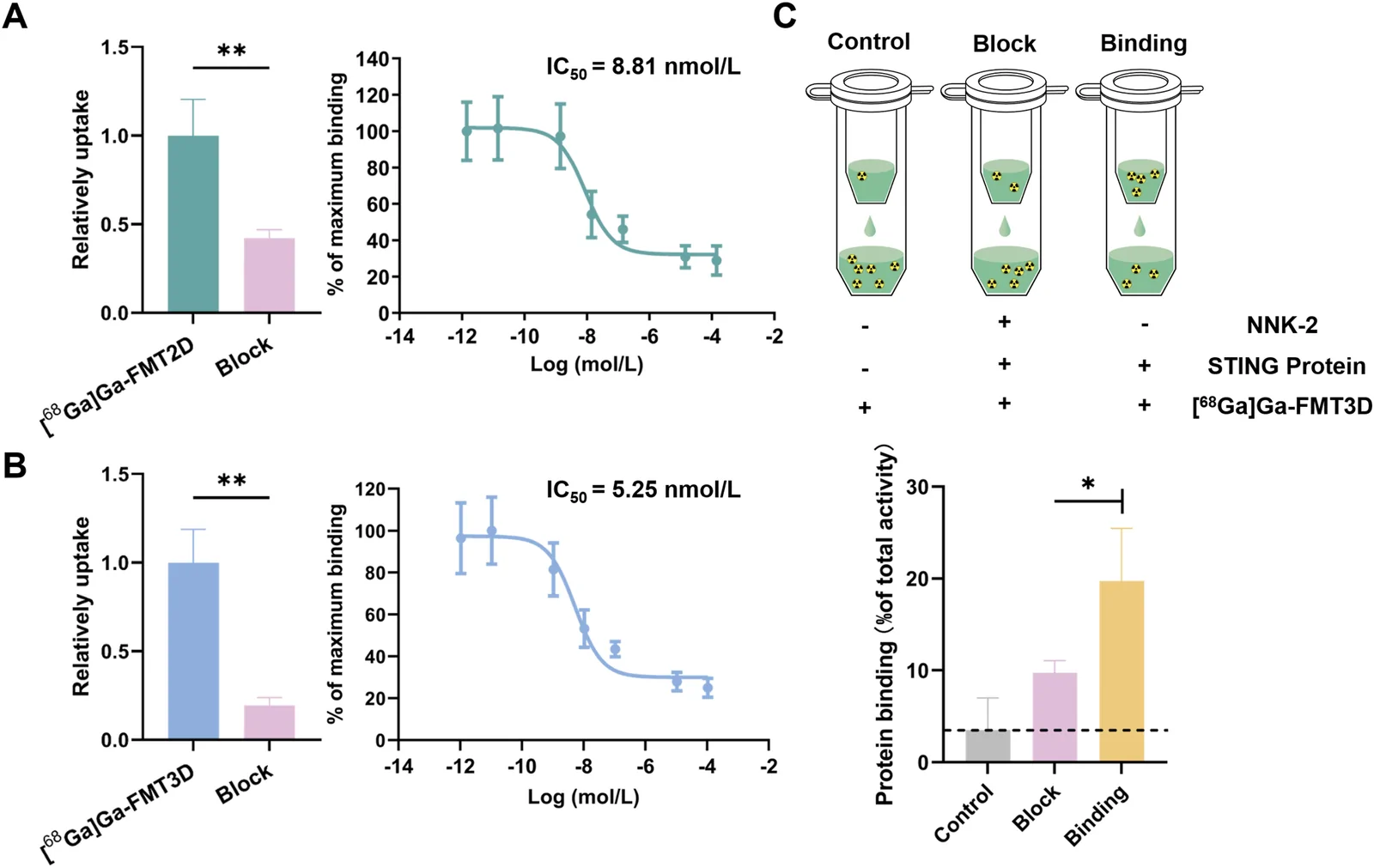
Cell uptake and competition assays of [^68^Ga]Ga-FMT2D (A) and [^68^Ga]Ga-FMT3D (B) in Raw 264.7 cells. In cell uptake assays, the radiotracers uptake in the normal group was normalized to 1, and the relative uptake was calculated as the ratio of the blocking group to the normal group. (C) Graphical representation of the centrifugal ultrafiltration assay and the binding rate of [^68^Ga]Ga-FMT3D with STING protein (^∗^*P* < 0.05, ^∗∗^*P* < 0.01).

A centrifugal ultrafiltration assay was initiated to further determine the specific binding ability of [^68^Ga]Ga-FMT3D to the STING protein. Expectedly, the binding group exhibited a higher protein binding rate of 19.74 ± 5.72%, which could be significantly inhibited by NNK-2 (9.74 ± 1.34%, ∗*P* < 0.05) addition (Fig. 4C). These results proved that [^68^Ga]Ga-FMT3D was a specific radiotracer that targeted STING.

### 2.6 PET imaging of [^68^Ga]Ga-FMT2D and [^68^Ga]Ga-FMT3D

As illustrated in Fig. 5, tumors were clearly visualized in B16F10 tumor-bearing mice at 0.5 h p.i. of [^68^Ga]Ga-FMT2D and [^68^Ga]Ga-FMT3D, with tumor uptake values of 2.80 ± 0.40%ID/g and 2.60 ± 0.10%ID/g, respectively. The tumor uptake values of the two radiotracers gradually increased over time, peaking at 1 h p.i. and gradually decreased (2.20 ± 0.20%ID/g for [^68^Ga]Ga-FMT2D at 2 h p.i., and 2.40 ± 0.06%ID/g for [^68^Ga]Ga-FMT3D at 2 h p.i.). Moreover, [^68^Ga]Ga-FMT3D exhibited superior imaging quality, particularly in terms of contrast, compared with [^68^Ga]Ga-FMT2D. Furthermore, dynamic PET imaging of [^68^Ga]Ga-FMT3D indicated that the uptake values in normal tissues such as the muscle, heart, and liver decreased over time (Supporting Information Fig. S14). To confirm the specificity of the two radiotracers, NNK-2 was selected as a competitor, the tumor uptake values of [^68^Ga]Ga-FMT2D and [^68^Ga]Ga-FMT3D were significantly reduced at 1 h p.i. (∗∗*P* < 0.01 and ∗∗∗∗*P* < 0.0001, respectively), indicating the STING-specific tumor uptake of these radiotracers.

**Figure 5:**
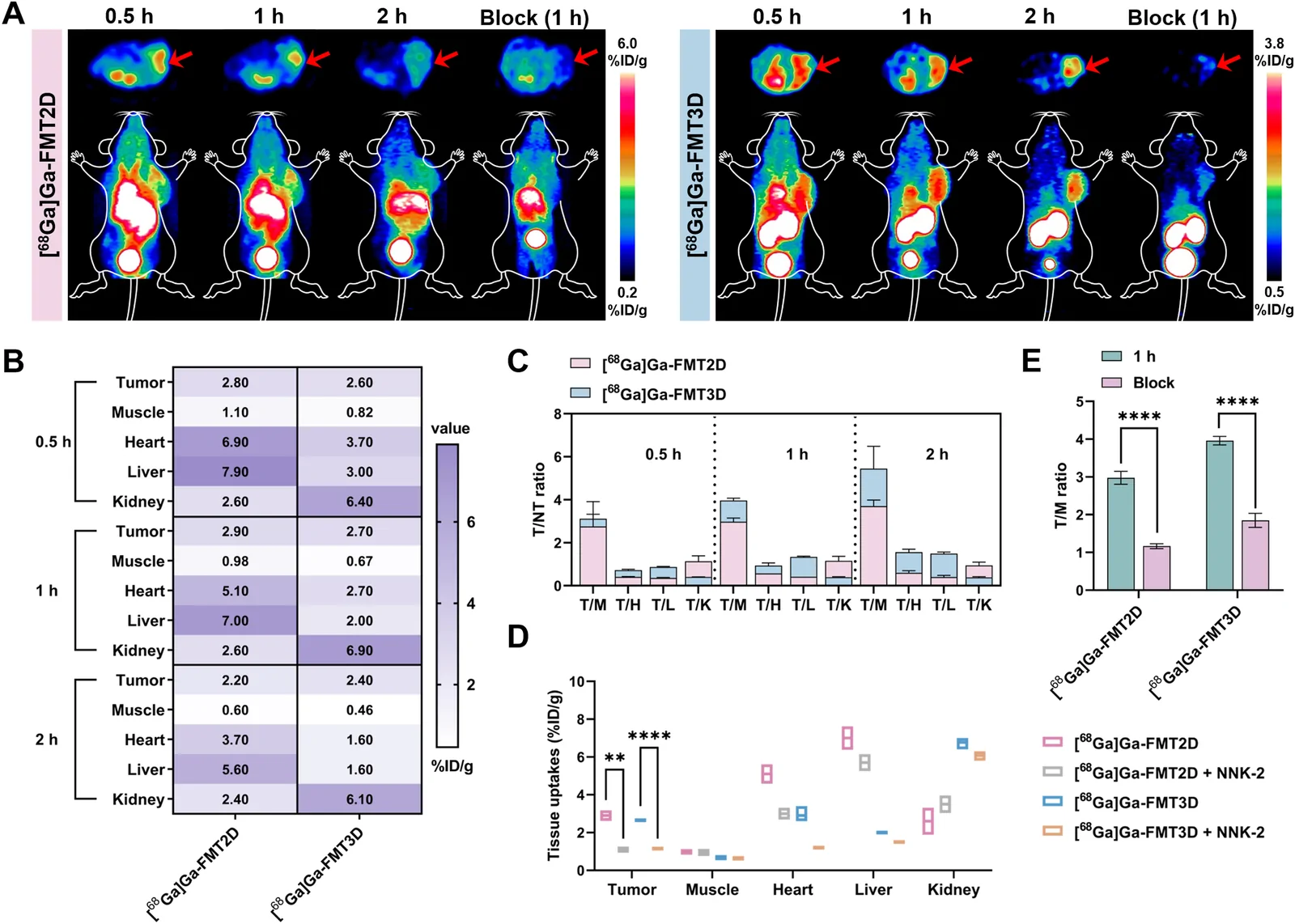
Small animal PET imaging of [^68^Ga]Ga-FMT2D and [^68^Ga]Ga-FMT3D in B16F10 tumor-bearing mice. (A) Representative MIP images at different time points. In the blocking group, mice were pre-injected with NNK-2. The uptakes of major organs (B) and tumor-to-normal tissue ratio (C) of radiotracers were derived from PET images at different time points. The uptakes of major organs (D) and tumor-to-muscle ratio (E) of radiotracers with or without NNK-2 at 1 h p.i. T: tumor, M: muscle, H: heart (including contents), L: liver, K: kidney (^∗∗^*P* < 0.01, ^∗∗∗∗^*P* < 0.0001).

PET imaging of [^68^Ga]Ga-FMT2D and [^68^Ga]Ga-FMT3D were also performed in 4T1 and PANC02 tumor-bearing mice (Supporting Information Fig. S15). The tumor uptake values of [^68^Ga]Ga-FMT2D and [^68^Ga]Ga-FMT3D in PANC02 tumor-bearing mice at 1 h p.i. were significantly higher than that in 4T1 tumor-bearing mice. This finding aligned with STING immunofluorescence data across different tumors. As shown in the immunofluorescence results in Supporting Information Fig. S16, the expression levels of the STING protein were the highest in B16F10, followed by PANC02, and the lowest in 4T1. Thus, the two radiotracers could visualize the STING level of the tumor *in vivo*.

### 2.7 Biodistribution and estimated absorbed radiation

The biodistribution data for [^68^Ga]Ga-FMT2D and [^68^Ga]Ga-FMT3D in B16F10 tumor-bearing mice at 1 h p.i. are summarized in Fig. 6. The tissue uptake values of the major organs are detailed in Supporting Information Table S2. The tumor uptake values of [^68^Ga]Ga-FMT2D and [^68^Ga]Ga-FMT3D were 6.49 ± 0.79%ID/g and 5.56 ± 0.56%ID/g, respectively. As shown in Supporting Information Table S3, the corresponding T/M ratios were 4.43 ± 0.81 and 6.12 ± 1.48, respectively. In normal tissues, [^68^Ga]Ga-FMT2D mainly accumulated in the liver, lung, and blood, with uptake values of 22.10 ± 6.98%ID/g, 19.32 ± 4.37%ID/g, and 19.46 ± 3.42%ID/g, respectively. This finding indicated that [^68^Ga]Ga-FMT2D was primarily metabolized *via* the hepatobiliary pathway and ultimately excreted through the intestines. Conversely, [^68^Ga]Ga-FMT3D mainly localized in the kidneys (67.91 ± 6.64%ID/g) and was then metabolized through the renal pathway. These results align with PET data. Moreover, the blood elimination half-lives (*t*_1/2_) of [^68^Ga]Ga-FMT2D and [^68^Ga]Ga-FMT3D were 89.9 and 62.1 min, respectively (Fig. 6C).

**Figure 6:**
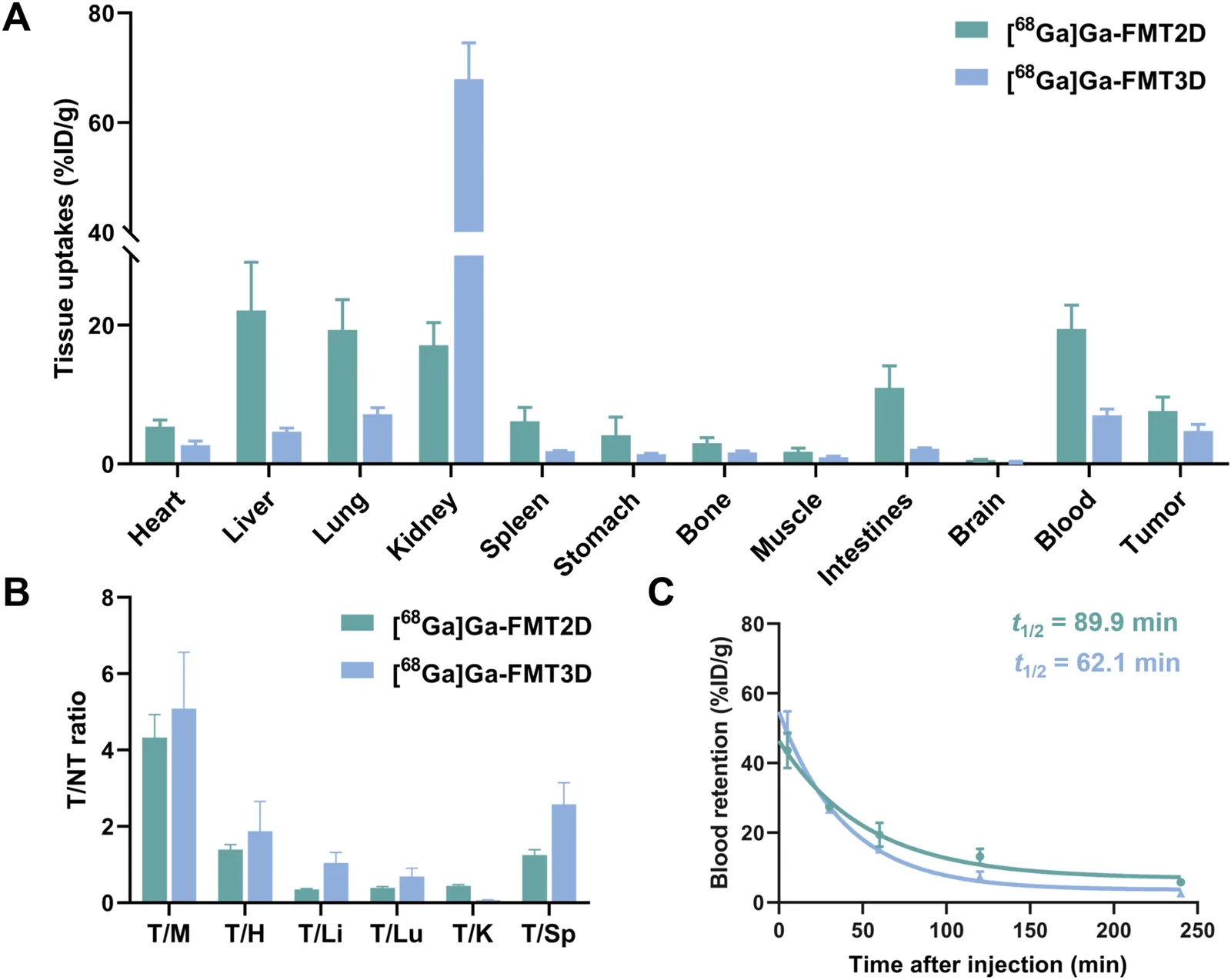
(A) Biodistribution results of [^68^Ga]Ga-FMT2D and [^68^Ga]Ga-FMT3D in B16F10 tumor-bearing mice at 1 h p.i. Tumor-to-normal tissue ratio (B) and time-dependent blood clearance (C) of [^68^Ga]Ga-FMT2D and [^68^Ga]Ga-FMT3D derived from biodistribution. T: tumor, M: muscle, H: heart (including contents), Li: liver, Lu: lung, K: kidney, Sp: spleen.

Organ-specific absorbed doses (mSv/MBq) are detailed in Supporting Information Table S4. The kidney showed the highest dose (0.0227 mSv/MBq), followed by the LLI wall (0.0175 mSv/MBq) and lung (0.0149 mSv/MBq). The average effective dose was 0.00776 mSv/MBq. These values fall within safe clinical limits, demonstrating the potential of [^68^Ga]Ga-FMT3D for diagnostic imaging.

### 2.8 STING-targeted imaging reflects the response to chemotherapy

DOX, a widely used anthracycline, has been reported to activate the cGAS–STING pathway by inducing DNA damage. The potential of ^68^Ga-labeled macrocyclic peptides for imaging STING levels was further evaluated in B16F10 tumor-bearing mice with or without DOX pretreatment. The tumor uptake values substantially increased to 2.8 ± 0.13%ID/g for [^68^Ga]Ga-FMT3D after DOX therapy, compared with 2.1 ± 0.08%ID/g in the control group (∗∗∗*P* < 0.001) (Fig. 7B and C). *Ex vivo* IHC staining confirmed the changes in STING expression in the tumor after chemotherapy, which indicated that the DOX-treated mice had higher STING levels (Fig. 7D and E). As depicted in Fig. 7F, the tumor uptake from the PET images correlated well with the STING expression determined by *ex vivo* IHC ([^68^Ga]Ga-FMT3D, *r* = 0.911, *P* < 0.001). The Western blot results were also in agreement with the IHC data (∗∗∗∗*P* < 0.0001) (Fig. 7G). In addition, increased heart uptake of [^68^Ga]Ga-FMT3D was observed after DOX treatment, which can be attributed to doxorubicin-induced cardiotoxicity, a common adverse effect linked to activation of the cGAS–STING pathway.

**Figure 7:**
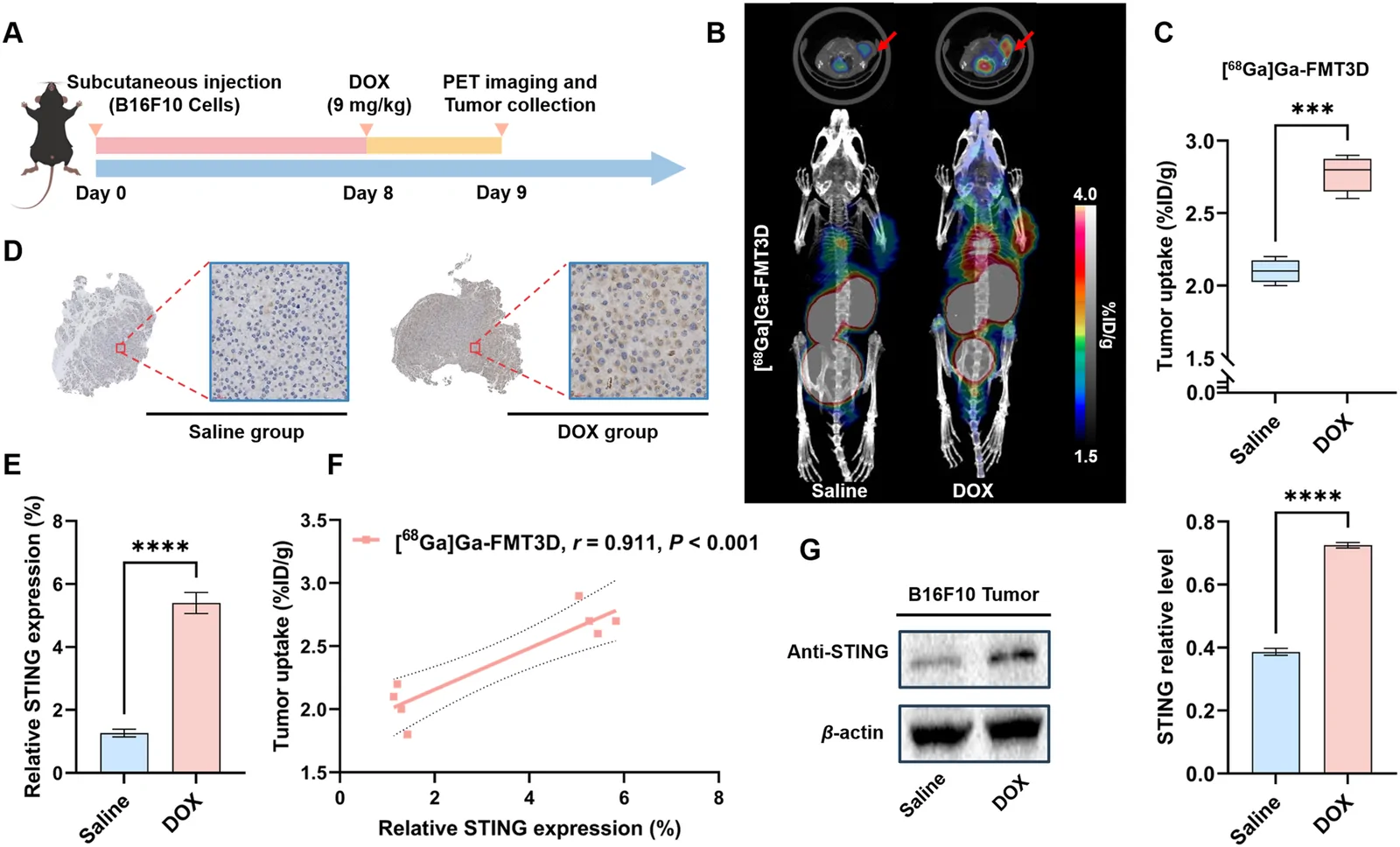
(A) Timeline of DOX injection and PET imaging. (B) Representative PET/CT images of B16F10 tumor-bearing mice with or without treated DOX on Day 9. (C) Quantification tumor uptake from PET results of [^68^Ga]Ga-FMT3D. Immunohistochemistry staining of STING in tumors (D) and quantification analysis (E). (F) Correlation plot of tumor uptake of radiotracers *vs* STING expression (IHC) on tumors from mice undergoing PET imaging. (G) Western blotting analysis.

## 3 Discussion

Recently, the cGAS–STING pathway has become one of the most vital signaling pathways in cancer immunotherapy^4^^,^^37^. The status of STING, as a key adaptor protein, is crucial for STING-related therapy, functioning as a biomarker for patient screening, predicting immune efficacy, and assessing prognosis^38^, ^39^, ^40^. Our previous study focused on the development of fluorine-18 and iodine-131 labeled STING agonists and inhibitors to visualize STING expression in tumors, and preclinical evaluations have yielded promising results^22^, ^23^, ^24^, ^25^, ^26^. Notably, all the aforementioned radiotracers exhibit considerable uptake in the gastrointestinal tract and gallbladder, which may be attributed to their high lipophilicity, potentially compromising imaging quality and restricting their clinical applications. Therefore, to further optimize the structure–activity relationship and identify lead structures better suited for the development of radiotracers, this study focuses on peptide-based scaffolds, particularly the modification of cyclic peptides. Cyclic peptides possess greater conformational rigidity than other structures, leading to improved *in vivo* stability and pharmacokinetics^41^^,^^42^.

As a new modality of ligands, cyclic peptides hold promise in the discovery of novel STING PET tracers, which has not yet been reported. Regarding radionuclides, gallium-68 is preferred in this study due to its ease of availability and optimal half-life. Additionally, gallium-68/lutetium-177 form an ideal theranostic pair and utilizd the same precusor, this study will also provide insights and precursor resources for future research on STING-targeted radioligand therapy. Therefore, in this study, cyclic peptide-based PET tracers, a series of ^68^Ga-labeled peptides, were developed and evaluated for targeting and imaging the STING protein *in vitro* and *in vivo*.

Initially, NNK-2, a reported STING-targeted macrocyclyic peptide with a *K*_d_ of 3.4 nmol/L, was selected as the lead compound^36^. To further explore the structure–activity relationship, amino acid substitutions at positions 2 and 9 of NNK-2 were optimized, as well as linker modifications, leading to the synthesis of four precursor compounds. Molecular docking results proposed that the modified ligands had higher docking scores than NNK-2, with ^nat^Ga-FMT3D showing the highest binding affinity for the STING protein. While computational assistance has facilitated radioprobe development, verification through biological experiments is still required. Four radiotracers were synthesized successfully, possessing high RCY, RCP, and molar activities. In this study, Raw 264.7 cells were chosen for the cell uptake and competition assays to assess the STING-targeting specificity of the radiotracers under high STING expression conditions. In contrast, the PET studies were designed to assess the *in vivo* STING-targeting ability of radiotracers within the tumor microenvironment. Therefore, B16F10 (melanoma), PANC02 (pancreatic cancer), and 4T1 (breast cancer) tumor models were established to examine radiotracers performance across different tumor types under immunocompetent conditions. PET imaging of B16F10 tumor-bearing mice showed that [^68^Ga]Ga-FMAD and [^68^Ga]Ga-FMTD exhibited suboptimal tumor uptake, indicating that position 9 modifications diminished the efficacy of the macrocyclic peptides in targeting STING. Conversely, [^68^Ga]Ga-FMT2D and [^68^Ga]Ga-FMT3D, modified at position 2, effectively identified STING protein expression in various tumor-bearing mouse models, which could be inhibited by the competitor NNK-2. *In vitro* cell and protein binding studies unveiled that the two radiotracers exhibited high affinities for STING. Further characterization and *in vivo* biodistribution studies revealed that [^68^Ga]Ga-FMT2D is primarily cleared through the hepatic-intestinal system, with relatively high uptake in the liver, lung, and intestines, which significantly compromises its tumor imaging quality throughout the body. Conversely, [^68^Ga]Ga-FMT3D was cleared through renal excretion and demonstrated superior pharmacokinetic properties, as evidenced by its higher T/M ratio. [^68^Ga]Ga-FMT3D also exhibited a shorter blood elimination half-life than [^68^Ga]Ga-FMT2D.

DOX, an anthracycline antibiotic, is one of the most widely prescribed chemotherapeutic agents for the treatment of numerous cancers^43^. Mechanistic studies have demonstrated that DOX induces mitochondrial DNA damage and release, which activates the cGAS–STING pathway, leading to enhanced antitumor immune responses and improved therapeutic outcomes, particularly when combined with chemotherapy and immunotherapy^44^, ^45^, ^46^, ^47^. The increased STING levels could be as a biomarker for monitoring DOX therapy efficacy. The [^68^Ga]Ga-FMT3D could be utilized for noninvasively assess the increase in STING protein expression in the tumor after DOX therapy. Strong STING expression, as detected by [^68^Ga]Ga-FMT3D, is associated with enhanced immune activation and improved therapeutic outcomes. In contrast, weak STING signals may indicate insufficient immune modulation, suggesting the potential benefit of combining immune checkpoint inhibitors (*e.g.*, PD-1/PD-L1 inhibitors) to enhance therapeutic efficacy.

Compared with previously reported STING-targeted radiotracers, [^68^Ga]Ga-FMT3D displays favorable pharmacokinetics and superior imaging quality, making it a strong candidate for clinical translation. While, high heart uptake of [^68^Ga]Ga-FMT2D/FMT3D in mice raises concerns about potential toxicity of radiotracers and their therapeutic viability as radiopharmaceuticals. To further address this concern, we performed hematoxylin and eosin (H&E) staining of heart tissues, which revealed no observable histopathological abnormalities after radiotracers administration, indicating that [^68^Ga]Ga-FMT3D did not induce detectable cardiotoxic effects (Supporting Information Fig. S17). Further structure−activity optimization is essential to enhance tumor uptake while minimizing renal retention. The development of noninvasive radioligands for visualizing the STING protein not only enhances our understanding of its role in immunotherapy but also provides a valuable tool for determining patients likely to benefit from targeted therapies, thereby informing precision treatment strategies. For instance, quantifying STING protein expression through PET imaging before administering STING agonists or in combination with immunotherapy can aid clinicians in optimizing drug timing and dosing, maximizing antitumor immune responses, and mitigating the negative regulation of the STING pathway. In addition, [^68^Ga]Ga-FMT3D could be applied beyond oncology, including the noninvasive assessment of chemotherapy-induced cardiotoxicity and the monitoring of STING activation in various inflammatory diseases and autoimmune disorders. Such applications would further broaden its potential clinical utility and provide valuable insights into STING-related pathophysiology.

## 4 Conclusions

In this study, first-in-class macrocyclic peptide-based PET tracers ([^68^Ga]Ga-FMAD, [^68^Ga]Ga-FMTD, [^68^Ga]Ga-FMT2D, and [^68^Ga]Ga-FMT3D) were developed and evaluated for *in vivo* imaging of STING expression in tumors. The high affinity and specificity, favorable pharmacokinetics, and comparable radiation dosimetry of [^68^Ga]Ga-FMT3D make it a promising tracer for PET imaging of STING status in clinical settings. This study provides a paradigm for synthesizing radiotracers for various imaging applications and improves radiotracer *in vivo* metabolism.

## 5 Experimental

### 5.1 Radiochemistry

Details of the reagents, chemicals, synthesis route of the precursors are described in the Supporting Information. For radiosynthesis, approximately 50 μg (21‒28 nmol) of labeling precursor dissolved in 1 mL of sodium acetate (0.25 mol/L in deionized water) was added to the 4 mL of ^68^GaCl_3_ (0.74‒1.11 GBq in 0.05 mol/L HCl) to adjust pH 4.0‒4.5. The reaction solution was heated to 85 °C for 15 min. After cooling the solution to room temperature (RT), the mixture was purified using a Sep-Pak C18 cartridge (WAT020515, Waters) with 1 mL of ethanol as the eluent. Nitrogen atmosphere was used to remove the ethanol at 60 °C. The radiochemical characterization was analyzed using radio-HPLC. The gradient condition was as follows: A was water with 0.1% TFA, B was acetonitrile with 0.1% TFA; 0‒20 min, 20%‒95% B; 21‒35 min, 20% B. The flow rate was 1 mL/min.

### 5.2 Docking simulation

The docking simulation were performed followed previous reported method. The structure of the STING (PDB:6DXL) was obtained from Protein Data Bank. All docking runs were performed using Glide module in Schrödinger Maestro software (version-2018). Using the protein preparation module, operations such as deleting extra chains, adding polar hydrogen atoms, and removing water molecules were performed sequentially on the STING protein structure under the OPLS4 force field. Subsequently, energy minimized was performed on the small molecule ligand to obtain multiple possible conformations. The 3D structures of ligands used for docking study were constructed by PyMol.

### 5.3 Cell culture and cell study

Raw 264.7 cells were cultured in Dulbecco’s Modified Eagle’s Medium (DMEM, Gibco) containing 10% fetal bovine serum (FBS, Gibco) and 100 U/mL penicillin and 100 μg/mL streptomycin at 37 °C in a humidified atmosphere of 5% carbon dioxide.

For the subsequent cell uptake experiment, Raw 264.7 cells were seeded into 24-well plates and cultured until 80% confluence per well. And then the growth medium was aspirated and washed three times with 0.5 mL of PBS. Each well received 0.5 mL of medium (without FBS) containing radiotracers (approximately 74 kBq). After incubation at 37 °C for 1 h, the medium was removed, and the cells were washed three times with 0.5 mL of PBS. Afterwards, 0.5 mL of NaOH (1 mol/L) was used to lyse the cells, and the contents were collected in a tube for counting by *γ*-counter. For the blocking assays, NNK-2 (10 μg/well) was selected as a blocking agent to co-incubated with radiotracers.

In competition assays, Raw 264.7 cells were incubated in 24-well plates for 24 h at a density of 80% confluence per well. And then the growth medium was aspirated and washed three times with 0.5 mL of PBS. Each well received 0.3 mL of medium (without FBS) containing radiotracers (approximately 74 kBq), while 0.1 mL of different concentrations of precursors (10^−11^ to 10^−5^ mol/L) was added to the blocked group (*n* = 4). After incubation at 37 °C for 1 h, the medium was removed, and the cells were washed three times with 0.5 mL of PBS. Afterwards, 0.5 mL of NaOH (1 mol/L) was used to lyse the cells, and the contents were collected in a tube for counting by *γ*-counter.

### 5.4 Protein binding study

The protein binding study was divided into three groups: a control group, a block group, and a binding group. Each group received 200 μL of radiotracers solution (approximately 74 kBq). 4 μg of STING protein was added to ultrafiltration tubes (OD010C34, PALL) for the other two groups, except for the control group. For the blocking group, 10 μg of NNK-2 was added for co-incubation. After incubation at 37 °C for 1 h, the ultrafiltration tubes from each group were centrifuged at 12,000 rpm for 15 min and then washed three times with 0.2 mL of water. Subsequently, the counts in the ultrafiltration tubes from each group were measured using *γ*-counter.

### 5.5 Tumor cells and animal models

B16F10 cell lines were purchased from the China Center for Type Culture Collection (CCTCC) and cultured with DMEM containing 10% FBS, 100 U/mL penicillin and 100 μg/mL streptomycin at 37 °C and 5% CO_2_.

C57BL/6J male mice (16‒17 g, 6‒7 weeks old) were purchased from the Experimental Animal Center of Xiamen University, and all animal care and studies strictly followed the guidelines of the Animal Care and Use Committee of the Laboratory Animal Center of Xiamen University (XMULAC20230307 and XMULAC20190157). The right upper limb of each mouse was subcutaneously injected with 5 × 10^6^ cells suspended in 100 μL of PBS to establish B16F10 subcutaneous xenograft models. The tumor grew until it reached 200‒300 mm^3^ for the subsequent experiment.

For the doxorubicin-induced group, 9 mg/kg of doxorubicin (DOX) was administered intraperitoneally to B16F10 tumor-bearing mice and induced for 24 h resulting in the upregulation of STING protein.

### 5.6 PET imaging

B16F10 tumor-bearing mice received a tail-vein injection of 100 μL radiotracers (approximately 7.4‒11.1 MBq) and underwent 5 min static imaging at 0.5, 1 and 2 h post-injection (p.i.). Dynamic PET images were acquired over 1 h p.i. of 7.4‒11.1 MBq [^68^Ga]Ga-FMT3D in B16F10 tumor-bearing mice. For blocking experiments, NNK-2 (1.5 mg/kg), used as a competitive agent, was injected into the tail vein of B16F10 tumor-bearing mice 20 min pre the radiotracers injection. The images were reconstructed using the 2D/3D ordered-subset expectation maximization (2D/3D OSEM) algorithm. Regions of interest (ROIs) were delineated and the uptake was expressed as the percentage injected dose per gram of tissue (%ID/g).

### 5.7 Biodistribution

B16F10 tumor-bearing mice received a tail-vein injection of 100 μL radiotracers (approximately 0.74 MBq) and were sacrificed 1 h p.i. for a biodistribution study. The organs of interest, including the heart, liver, lung, kidneys, spleen, stomach, bone, muscle, intestines, brain, blood and tumor, were separated and weighed. Subsequently, the radioactivity of each organ was measured using *γ*-counter and expressed as mean ± SD (*n* = 3). Blood samples were collected at 5, 30, 60, 120, and 240 min p.i., and their radioactivity were measured. The blood half-life was then calculated using DSA sofeware.

### 5.8 Statistical analysis

The GraphPad Prism 9.0 software was used for statistical analysis. Quantitative data are presented as mean ± standard deviation (SD), and the differences between groups were assessed using Student’s *t*-test or one-way ANOVA. *P* < 0.05 was considered statistically significant (∗*P* < 0.05, ∗∗*P* < 0.01, ∗∗∗*P* < 0.001, ∗∗∗∗*P* < 0.0001).

## Author contributions

Jingru Zhang: Conceptualization, Data curation, Formal analysis, Methodology, Writing-original draft. Lingxin Meng: Conceptualization, Data curation, Methodology, Writing-original draft. Huifeng Li: Methodology, Data curation, Formal analysis. Dongsheng Xia: Methodology, Formal analysis. Xiaobo Wang: Conceptualization, Formal analysis, Methodology. Rongqiang Zhuang: Conceptualization, Formal analysis, Methodology. Huanhuan Liu: Conceptualization, Methodology. Yesen Li: Conceptualization, Methodology, Writing-review & editing. Xianzhong Zhang: Conceptualization, Methodology, Supervision, Validation, Writing-review & editing. Jianyang Fang: Conceptualization, Formal analysis, Methodology, Writing-original draft, Writing-review & editing. Zhide Guo: Conceptualization, Formal analysis, Methodology, Supervision, Validation, Writing-original draft, Writing-review & editing.

## Conflicts of interest

The authors have no conflicts of interest to declare.

## Appendix A Supporting information

The following is the Supporting Information to this article:
