# Supplementary material for: Development of 68Ga-labeled macrocyclic peptide for targeting stimulator of interferon genes
Source: Acta Pharm Sin B. 2026 Mar 11;16(11):7508–18. doi: 10.1016/j.apsb.2026.03.010 (PMC13729525; doi:10.1016/j.apsb.2026.03.010)
Supplement: Supplementary file 1 [file mmc1.pdf]

Supporting Information for

Original article

Development of ^{68}Ga-labeled macrocyclic peptide for targeting stimulator of interferon genes

Jingru Zhang[a,†], Lingxin Meng[a,†], Huifeng Li[a], Dongsheng Xia[a], Xiaobo Wang[f], Rongqiang Zhuang[a], Huanhuan Liu[a], Yesen Li[c,*], Xianzhong Zhang[e,*], Jianyang Fang[a,b,*], Zhide Guo[a,c,d,*]

[a] *State Key Laboratory of Vaccines for Infectious Diseases, Xiang An Biomedicine Laboratory, National Innovation Platform for Industry-Education Integration in Vaccine Research, Center for Molecular Imaging and Translational Medicine, School of Public Health, Xiamen University, Xiamen 361102, China*

[b] *School of Medical Imaging, Fujian Medical University, Fuzhou 350122, China*

[c] *Department of Nuclear Medicine, The First Affiliated Hospital of Xiamen University, School of Medicine, Xiamen University, Xiamen 361003, China*

[d] *Zhangzhou Affiliated Hospital of Fujian Medical University, Zhangzhou 363000, China*

[e] *Theranostics and Translational Research Center, National Infrastructures for Translational Medicine, Institute of Clinical Medicine & Department of Nuclear Medicine, Peking Union Medical College Hospital, Chinese Academy of Medical Sciences & Peking Union Medical College, Beijing 100730, China*

[f] *Department of Nuclear Medicine and State Key Laboratory of Holistic Integrative Management of Gastrointestinal Cancers, Xijing Hospital, Fourth Military Medical University, Xi'an 710032, China*

Received 3 November 2025; received in revised form 21 January 2026; accepted 7 February 2026

[†]These authors made equal contributions to this work.

[*]Corresponding authors.

E-mail addresses: gzd666888@xmu.edu.cn (Zhide Guo), fangjy8228@fjmu.edu.cn (Jianyang Fang), zhangxzh@pumch.cn (Xianzhong Zhang), leeyesen@gmail.com (Yesen Li)

Contents Page

Figure S1 Synthesis routes of the precursors FMAD and FMTD P. S8

Figure S2 Synthesis routes of the precursors FMT2D and FMT3D P. S9

Figure S3 HPLC analysis of the chemical purities of FMAD, FMTD, FMT2D and FMT3D. P. S10

Figure S4 ESI-MS spectrum of FMT2. P. S11

Figure S5 ESI-MS spectrum of FMAD. P. S12

Figure S6 ESI-MS spectrum of FMTD. P. S13

Figure S7 ESI-MS spectrum of FMT2D. P. S14

Figure S8 ESI-MS spectrum of FMT3D. P. S15

Figure S9 Structure of [^{68}Ga]Ga-FMAD, [^{68}Ga]Ga-FMTD, [^{68}Ga]Ga-FMT2D, and [^{68}Ga]Ga-FMT3D. P. S16

Figure S10 HPLC analysis of the radiochemical purity of [^{68}Ga]Ga-FMAD, [^{68}Ga]Ga-FMTD, [^{68}Ga]Ga-FMT2D, and [^{68}Ga]Ga-FMT3D. P. S17

Figure S11 *In vitro* stabilities of [^{68}Ga]Ga-FMAD and [^{68}Ga]Ga-FMTD in PBS or mouse serum. P. S18

Figure S12 *In vitro* stabilities of [^{68}Ga]Ga-FMT2D and [^{68}Ga]Ga-FMT3D in PBS or mouse serum. P. S19

Figure S13 Metabolic stabilities of [^{68}Ga]Ga-FMT3D in the urine or blood of C57BL/6J male mice. P. S20

Figure S14 Dynamic PET imaging, tumor-to-muscle ratio and tissue uptake of [^{68}Ga]Ga-FMT3D in B16F10 tumor-bearing mice. P. S21

Figure S15 PET imaging and tissues uptake of [^{68}Ga]Ga-FMT2D and [^{68}Ga]Ga-FMT3D at 1 h p.i. in 4T1 and PANC02 tumor-bearing mice. P. S22

Figure S16 Representative immunofluorescence images of different tumor types. P. S23

Figure S17. H&E staining of heart tissues after [^{68}Ga]Ga-FMT3D administration. P. S24

Table S1 Characteristics of [^{68}Ga]Ga-FMAD, [^{68}Ga]Ga-FMTD, [^{68}Ga]Ga-FMT2D, and [^{68}Ga]Ga-FMT3D. P. S25

Table S2 Biodistribution results of [^{68}Ga]Ga-FMT2D and [^{68}Ga]Ga-FMT3D in B16F10 tumor-bearing mice at 1 h p.i. P. S26

Table S3 Tumor-to-normal tissue ratio of [^{68}Ga]Ga-FMT2D and [^{68}Ga]Ga-FMT3D in B16F10 tumor-bearing mice at 1 h p.i. derived from biodistribution results. P. S27
Table S4. Estimated absorbed radiation dosimetry for [^{68}Ga]Ga-FMT3D in different organs (mSv/MBq). P. S28

1. Experimental

1.1. Reagents and instruments

All of the chemical reagents were obtained from commercial suppliers. NNK-2, FMA, FMT and FMT2 were custom synthesized by Nanchang Tanzhen Biotechnology co., ltd. DOTA-NHS was purchased from Xian Ruixi Biological Technology Co., Ltd. (China). Mass spectrometry (MS) was recorded on a Q Exactive LC-MS system (Waters, USA). $^{68}GaCl_3$ was obtained from the ^{68}Ge–^{68}Ga generator (ITM). Dionex Ulti-Mate 3000 high-pressure liquid chromatography (Thermo Scientific, USA) was equipped with an Elysia Raytest Gabi Star *γ*-radiation detector. Analytical C-18 reversed-phase HPLC column (4.6 mm×250 mm, 5 μm, 120 Å, Thermo) was used for the purity analysis. Radioactivity counts were examined with an WIZARD 2480 automated *γ*-counter (Perkin-Elmer, USA). PET/CT imaging was performed with an Inveon scanner (Siemens, USA).

1.2. Chemistry

Synthesis of FMAD. A mixture of FMA (4.4 mg, 3 μmol), DOTA-NHS (5.0 mg, 12 μmol), 20 μL of DIPEA, and 500 μL of DMF was placed into a 1.5 mL centrifugation tube. Then the centrifugation tube was agitated in an oscillator for 8 hours at room temperature (RT). The FMAD was segregated using semi-preparative HPLC with UV detection at 254 nm and obtained after lyophilization (2.7 mg, 48%). The retention time (R_t) of FMAD on HPLC was 9.62 min. ESI-MS calculated for $C_{93}H_{115}N_{19}O_{21}S$: requires 1866.83, found $[M+H]^+$ = 1867.25, $[M +2H]^{2+}$ = 934.12.

Synthesis of FMTD. A mixture of FMT (4.9 mg, 3 μmol), DOTA-NHS (5.0 mg, 12 μmol), 20 μL of DIPEA, and 500 μL of DMF was placed into a 1.5 mL centrifugation tube. Then the centrifugation tube was agitated in an oscillator for 8 h at RT. The FMTD was segregated using semi-preparative HPLC with UV detection at 254 nm and obtained after lyophilization (3.5 mg, 58%). The R_t of FMTD on analytical HPLC was 10.47 min. ESI-MS calculated for $C_{99}H_{126}N_{20}O_{24}S$: requires 2011.90, found $[M +2H]^{2+}$ = 1006.66.

Synthesis of FMT2D. A mixture of FMT2 (4.0 mg, 3 μmol), DOTA-NHS (5.0 mg, 12 μmol), 20 μL of DIPEA, and 500 μL of DMF was placed into a 1.5 mL centrifugation tube. Then the centrifugation tube was agitated in an oscillator for 8 h at RT. The FMT2D was segregated using semi-preparative HPLC with UV detection at 254 nm and obtained after lyophilization (2.9 mg, 55%). The R_t of FMT2D on analytical HPLC

was 11.32 min. ESI-MS calculated for $C_{89}H_{108}N_{18}O_{19}S$: requires 1764.78, found $[M+2H]^{2+}$= 883.19.

Synthesis of FMT3D. A mixture of FMT2 (4.0 mg, 3 μmol), F1 (12 μmol), 500 μL of Phosphate buffer (0.5 M, pH=9.0), and 500 μL of DMF was placed into a 1.5 mL centrifugation tube. Then the centrifugation tube was agitated in an oscillator for 8 hours at 50 °C. The FMT3D was segregated using semi-preparative HPLC with UV detection at 254 nm and obtained after lyophilization (3.5 mg, 49%). The R_t of FMT2D on analytical HPLC was 10.51 min. ESI-MS calculated for $C_{93}H_{115}N_{19}O_{21}S$: requires 2376.00, found $[M+2H]^{2+}$= 1188.27. $[M+3H]^{3+}$ =792.84.

1.3. Octanol/water partition coefficient (Log P)

The Octanol/water partition coefficient of radiotracers were measured 0.1 mL of radiotracers solution (approximately 0.74 MBq) was added to a centrifuge tube containing 0.9 mL phosphate buffered saline (pH=7.4) and 1 mL of 1-octanol. After vortex shaking for 2 min, the well-mixed solution was left to stand for another 3 min. Then the solution was centrifuged at 10,000 rpm for 3 min to partition the layers. Subsequently, 0.1 mL (n=3) of organic and aqueous layers were taken for radioactivity count determination by γ-counter to calculate Log P. P = (activity in 1-octanol) / (activity in the aqueous layer).

1.4. Stability Study

For the *in vitro* stability study, the radiotracers were incubated at 37 °C for 30, 60 and 120 min with mouse serum or PBS, respectively. Acetonitrile (0.5 mL) was added to deplete plasma proteins from the serum. After filtering the supernatant through a 0.22 μm filter, the resulting solution was subjected to analysis using radio-HPLC. The metabolic stabilities of the radiotracers were evaluated in C57BL/6J male mice at 30 and 60 min post-injection (p.i.). At each time point, the mice were sacrificed to collect blood and urine samples. The blood samples were centrifuged at 12,000 rpm for 5 min, and the supernatants were collected to obtain serum. Subsequently, acetonitrile was added to the serum and urine samples to precipitate proteins, and the mixtures were then passed through a 0.22 μm filter, respectively. The radiochemical purity (RCP) of radiotracers were assessed by radio-HPLC.

1.5. Western Blot

After PET imaging, the tumor samples were isolated and lysed in RIPA lysate buffer containing complete protease inhibitors and phosphatase inhibitor cocktails on ice. The

concentration of proteins was determined by the BCA Protein Assay Kit (P0010, Beyotime). Protein samples (20 μg) were separated on 10% SDS-polyacrylamide gels and transferred to the polyvinylidene fluoride membrane (Millipore). Membranes were blocked in 5% milk solution at RT for 1 h and then incubated with an anti-STING rabbit antibody (Cat# 13674, Cell Signaling Technology) and anti-*β*-actin antibody (BM0627, Boster) at 4 °C overnight. The next day, incubated with HRP-conjugated Goat Anti-Rabbit IgG (D110058-0100, Sangon Biotech) and HRP-conjugated Goat Anti-Mouse IgG (D110087-0100, Sangon Biotech) at RT for 2 h, respectively. Protein bands were visualized with Bio-Rad ChemiDoc™ XRS System.

1.6. Immunohistochemistry

After the last *in vivo* PET imaging scan, tumor samples were collected and sectioned into 4 μm slices. After antigen retrieval, tumor sections were blocked with 3% hydrogen peroxide solution at RT in the dark for 25 min, followed by blocking with 3% BSA solution at RT for 30 min. After carefully discarding the blocking solution, the tumor sections were incubated with an anti-STING rabbit antibody at 4 °C overnight. The next day, the tumor sections were incubated with HRP-conjugated Goat Anti-Rabbit IgG, followed by DAB chromogenic solution (G1212, Servicebio) and hematoxylin solution (G1004, Servicebio). Afterwards, the tumor sections were dehydrated and mounted. The images of the tumor sections were obtained using a light microscope (VM1, Motic).

1.7. Dosimetry Calculation

The radiation dosimetry of [^{68}Ga]Ga-FMT3D in adult females was extrapolated from preclinical biodistribution data obtained in normal ICR mice. Briefly, female ICR mice (n = 3 per time point) were intravenously injected with [^{68}Ga]Ga-FMT3D (3.7 MBq in 100 μL of saline). At predetermined time points post-injection (1, 5, 30, and 60 min), the mice were sacrificed, and major organs (including blood, heart, lungs, liver, spleen, kidneys, stomach, intestines, bone, and muscle) were excised, weighed, and their radioactivity measured using a *γ*-counter. The tissue uptake was expressed as the percentage of the injected dose per gram of tissue (%ID/g). Time-activity curves were plotted for each organ, and the corresponding areas under the curve (AUCs) were calculated using GraphPad Prism 9.0. Mouse biodistribution data were extrapolated to humans using mass and time scaling methods. The organ-absorbed doses and whole-body effective dose were subsequently estimated using OLINDA/EXM version 1.0 (Hermes Medical Solutions AB).

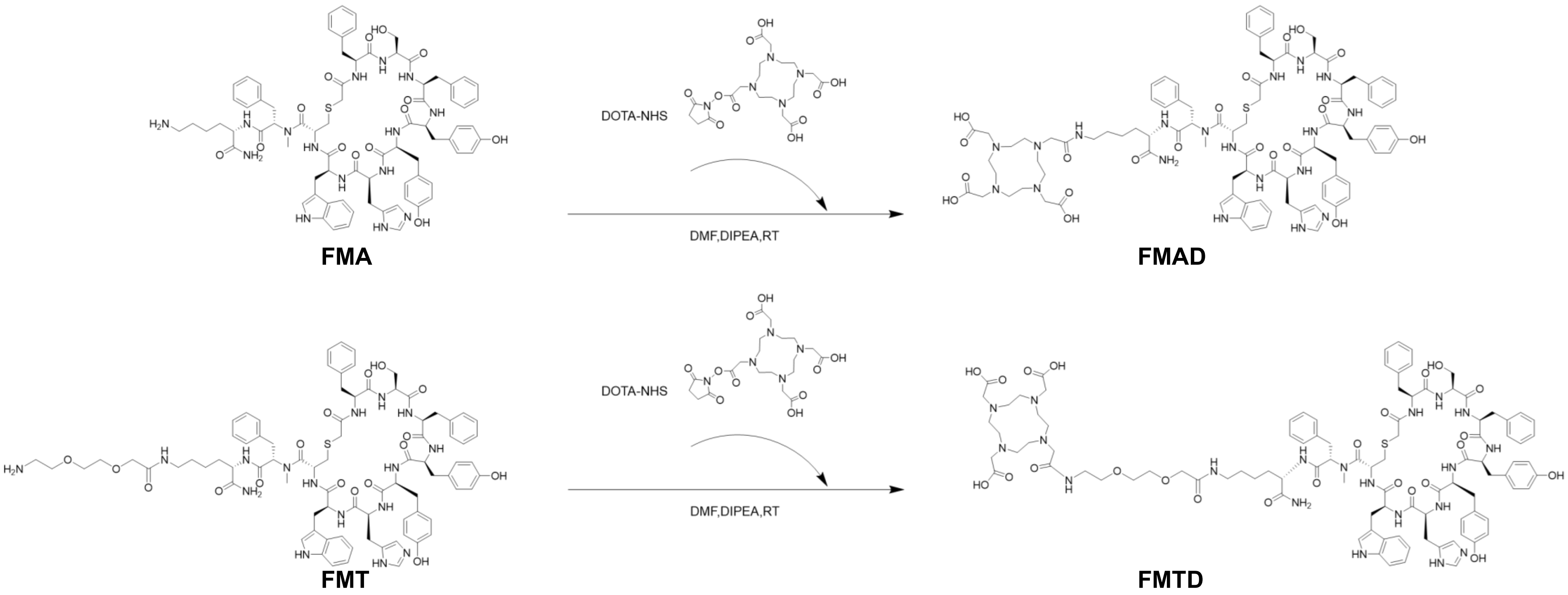

Figure S1 Synthesis routes of the precursors FMAD and FMTD.

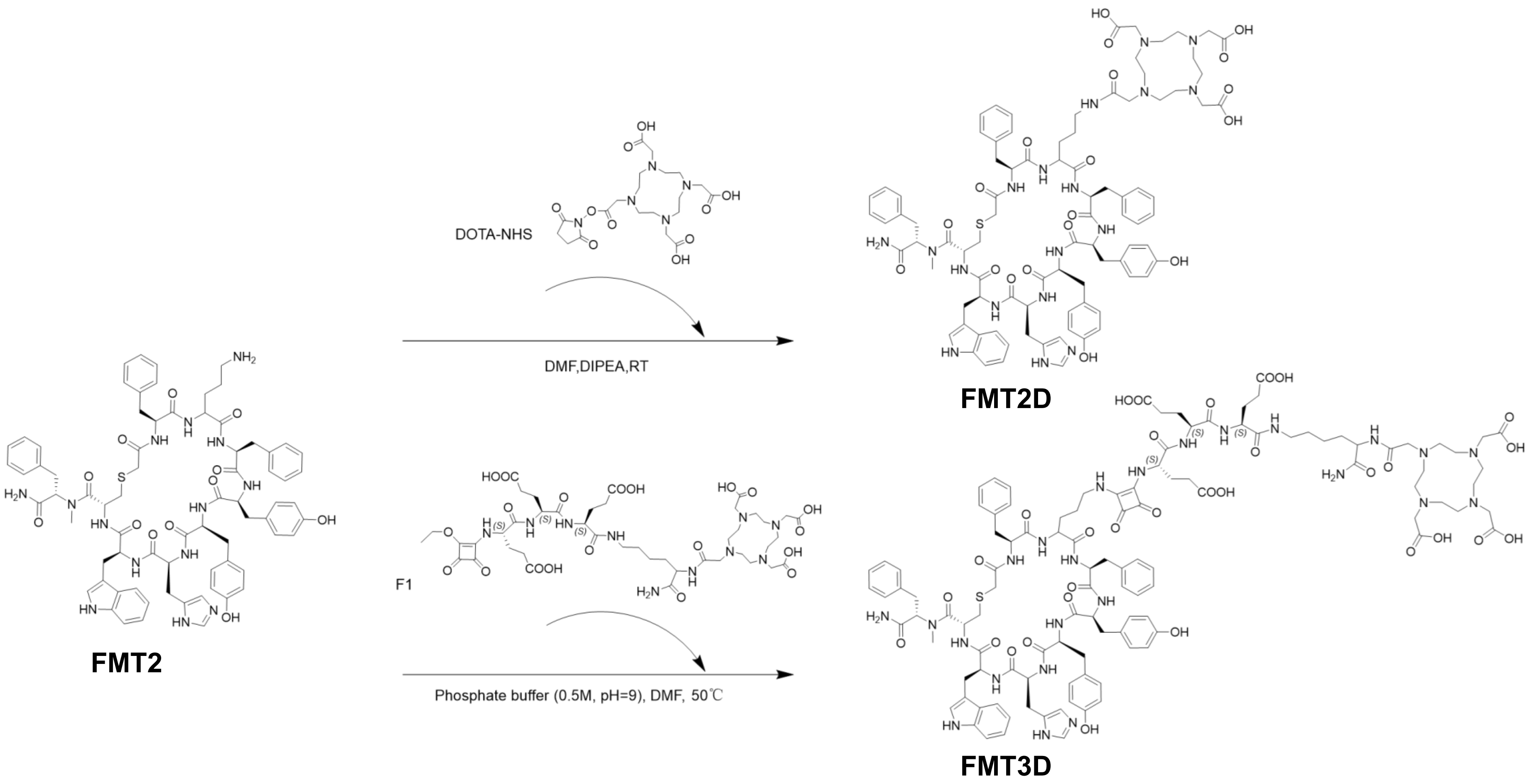

Figure S2 Synthesis routes of the precursors FMT2D and FMT3D.

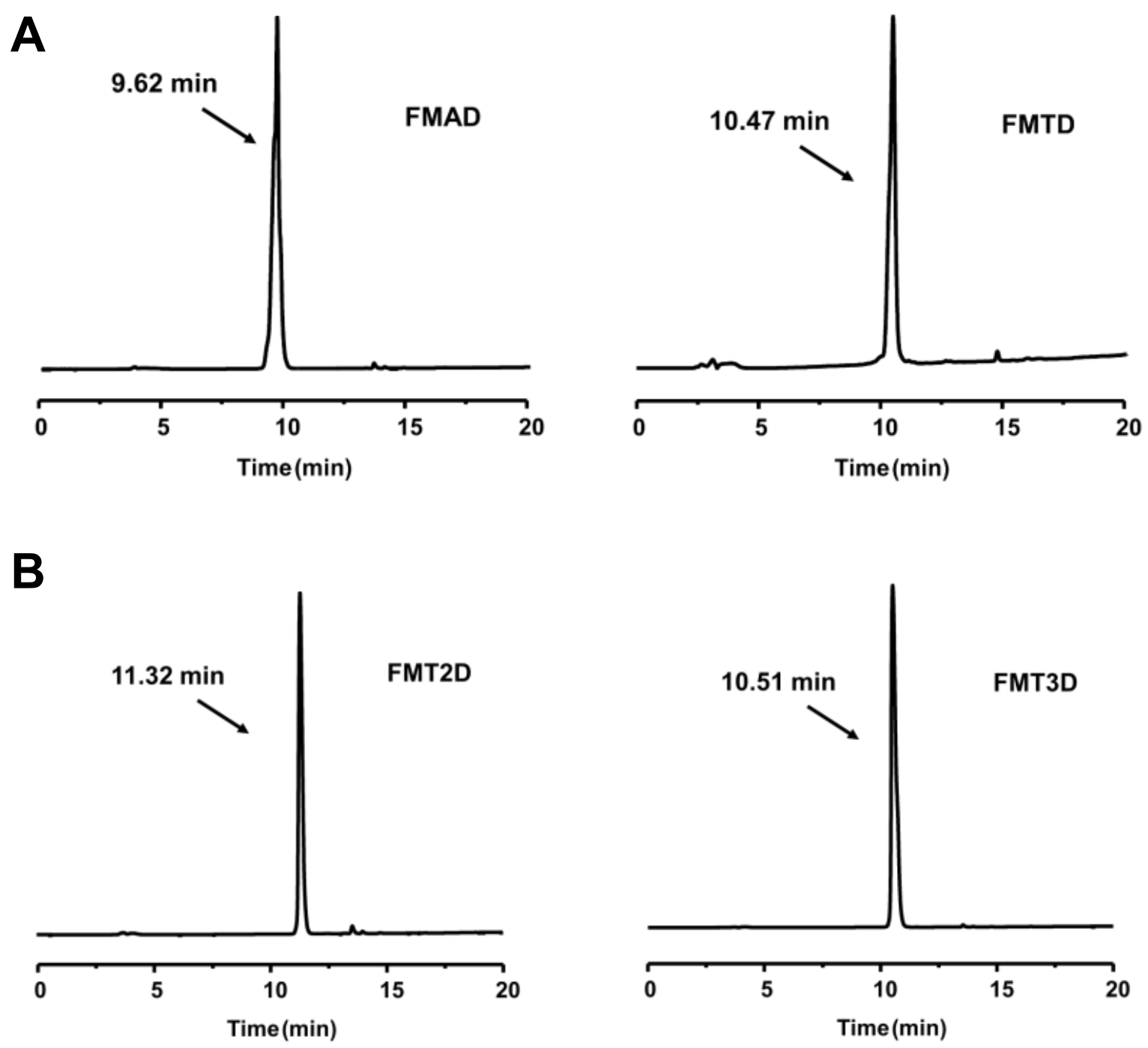

Figure S3 HPLC analysis of the chemical purities of FMAD, FMTD, FMT2D and FMT3D.

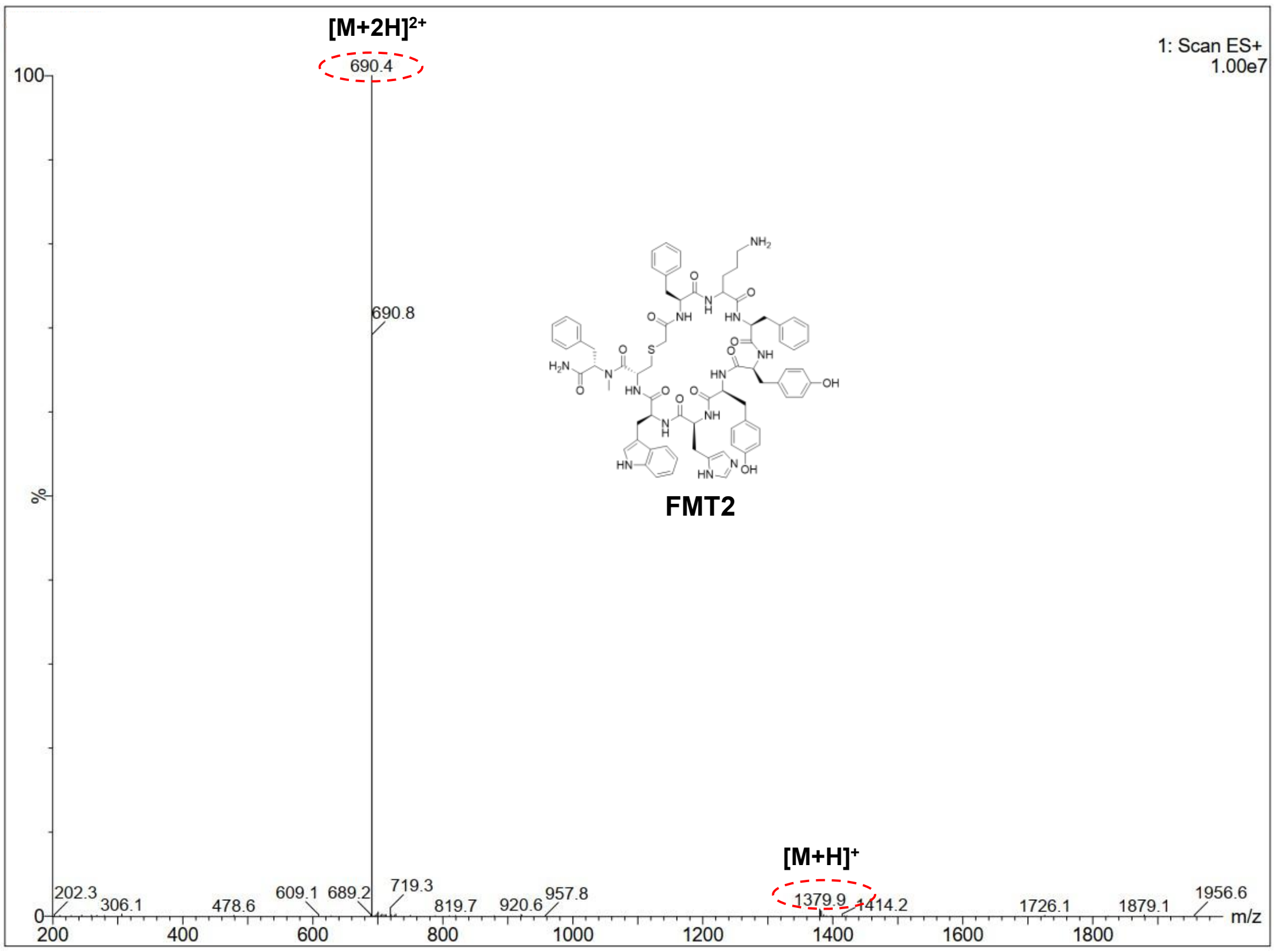

Figure S4 ESI-MS spectrum of FMT2.

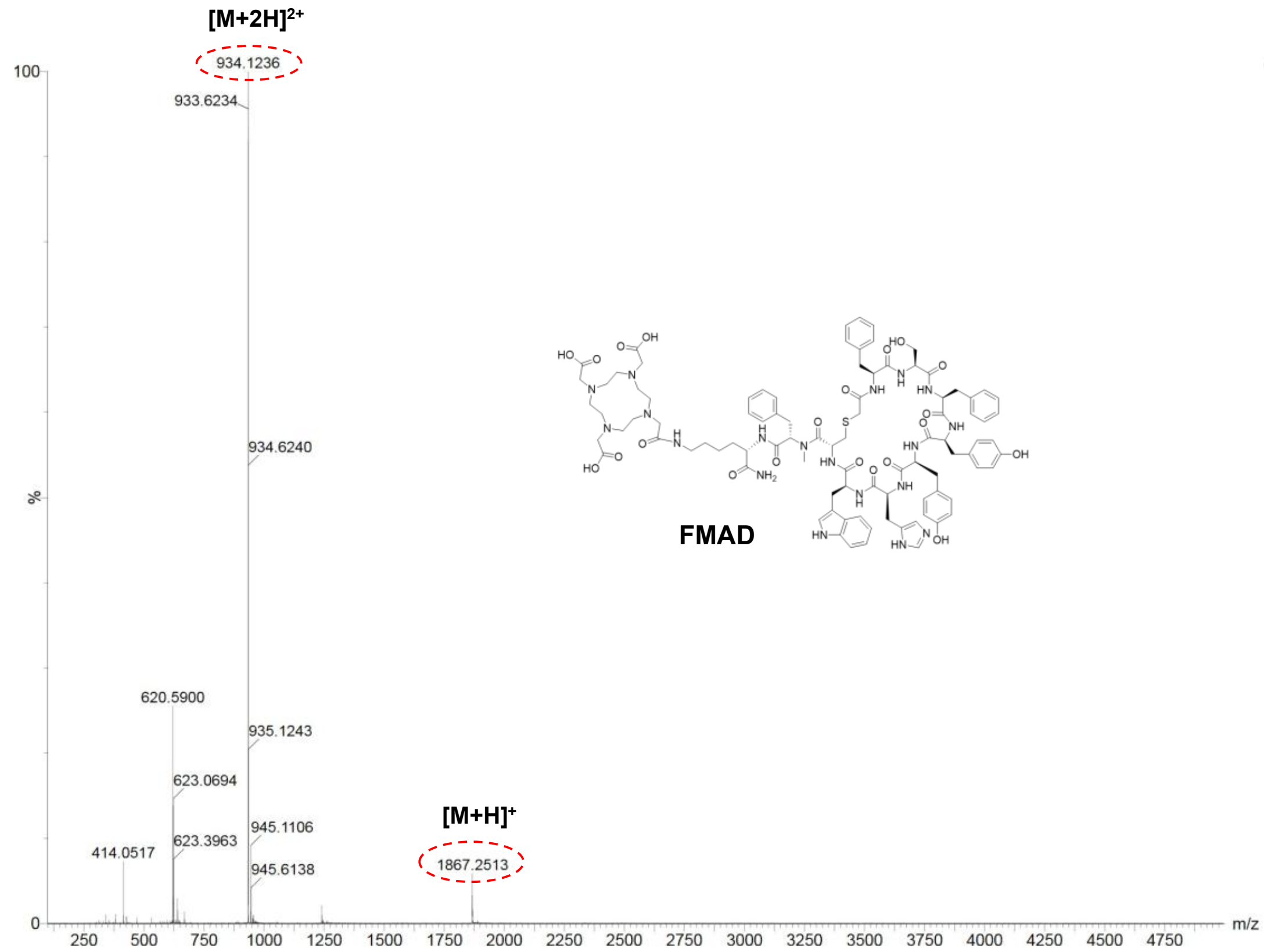

Figure S5 ESI-MS spectrum of FMAD.

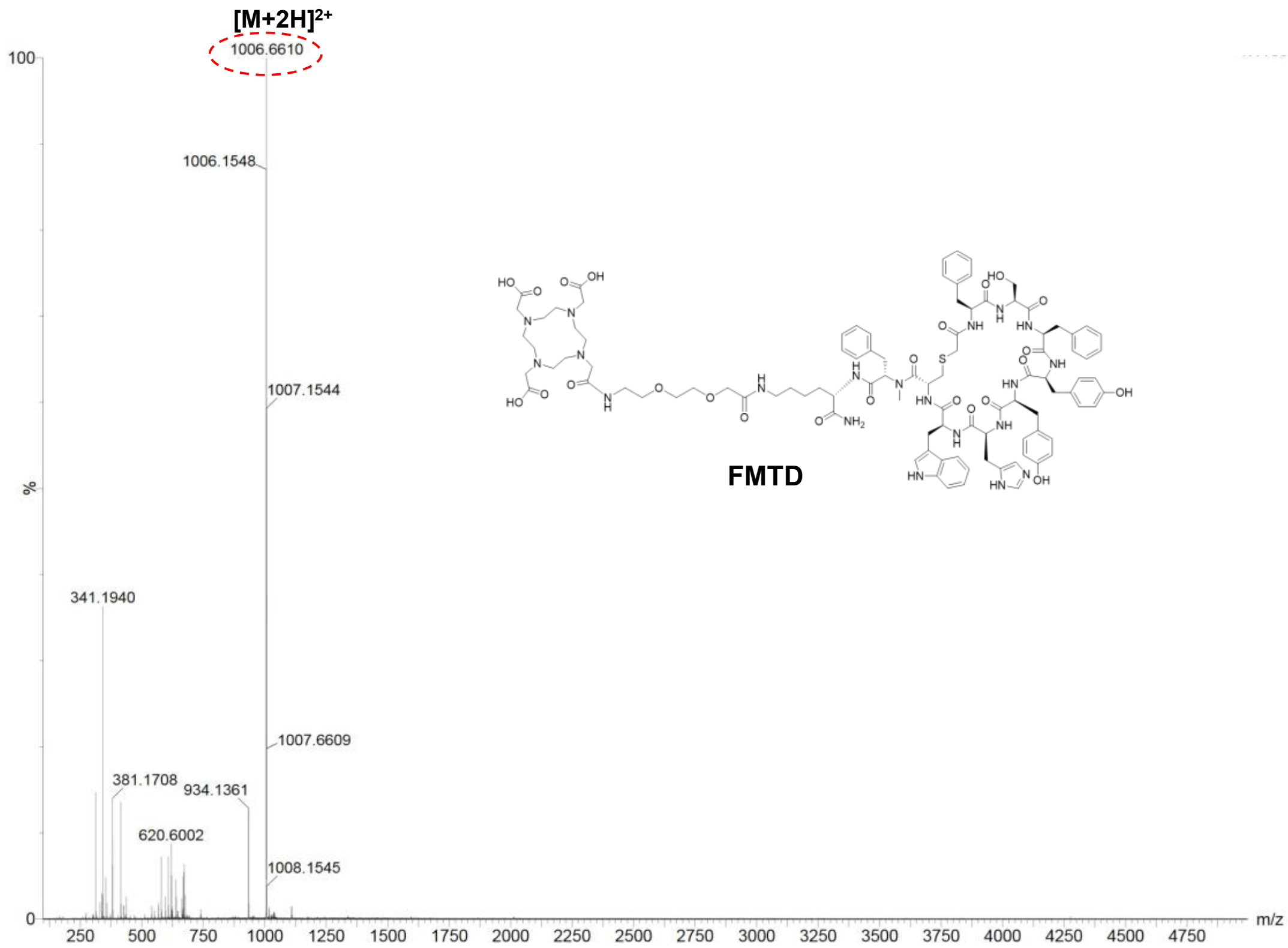

Figure S6 ESI-MS spectrum of FMTD.

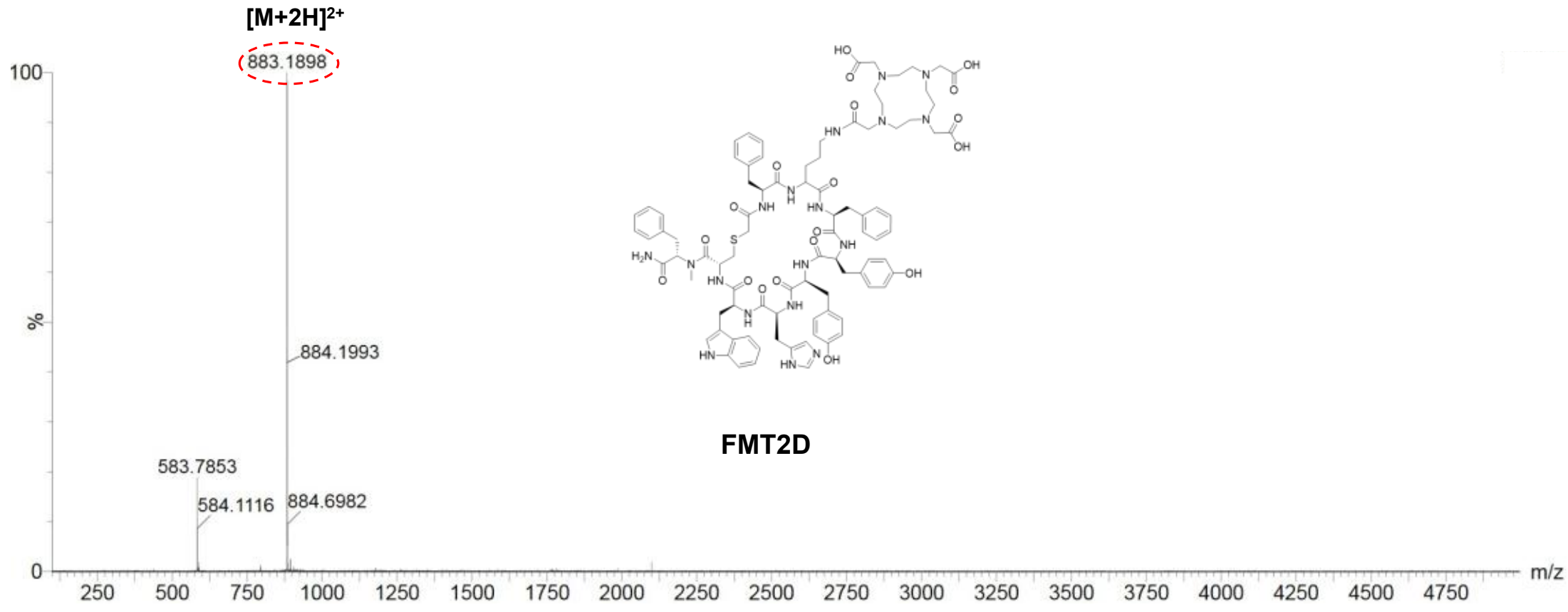

Figure S7 ESI-MS spectrum of FMT2D.

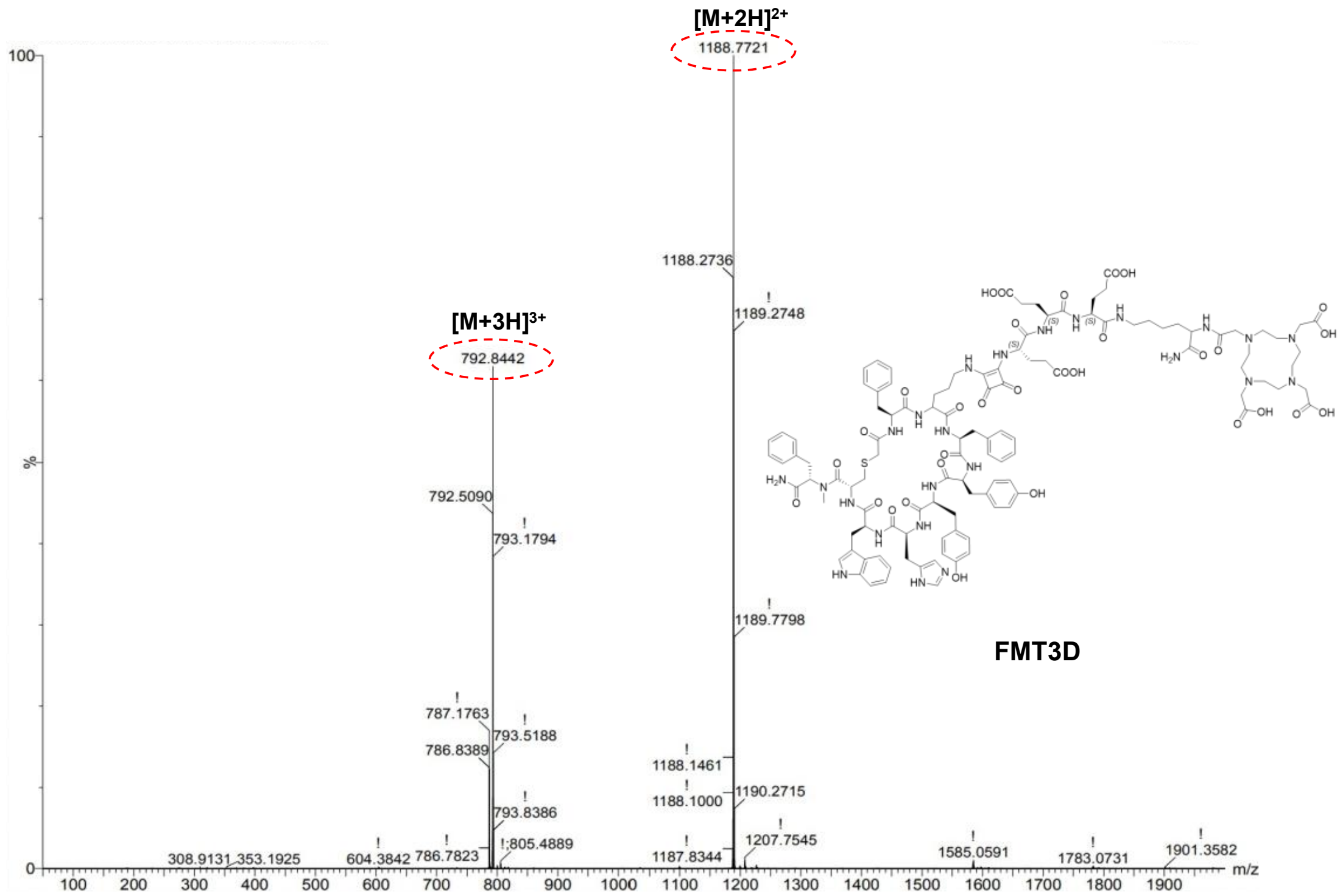

Figure S8 ESI-MS spectrum of FMT3D.

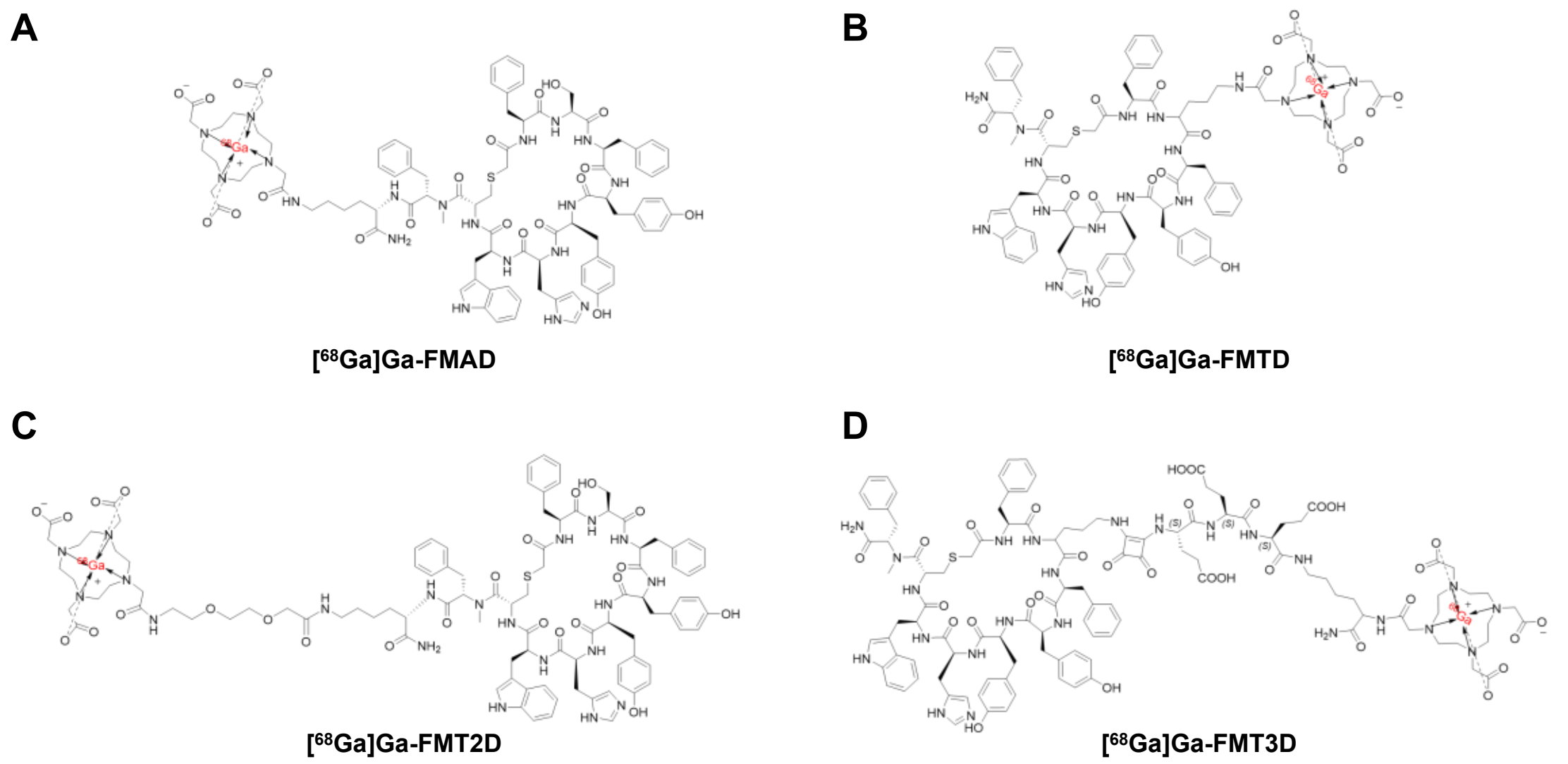

Figure S9 Structure of [^{68}Ga]Ga-FMAD, [^{68}Ga]Ga-FMTD, [^{68}Ga]Ga-FMT2D, and [^{68}Ga]Ga-FMT3D.

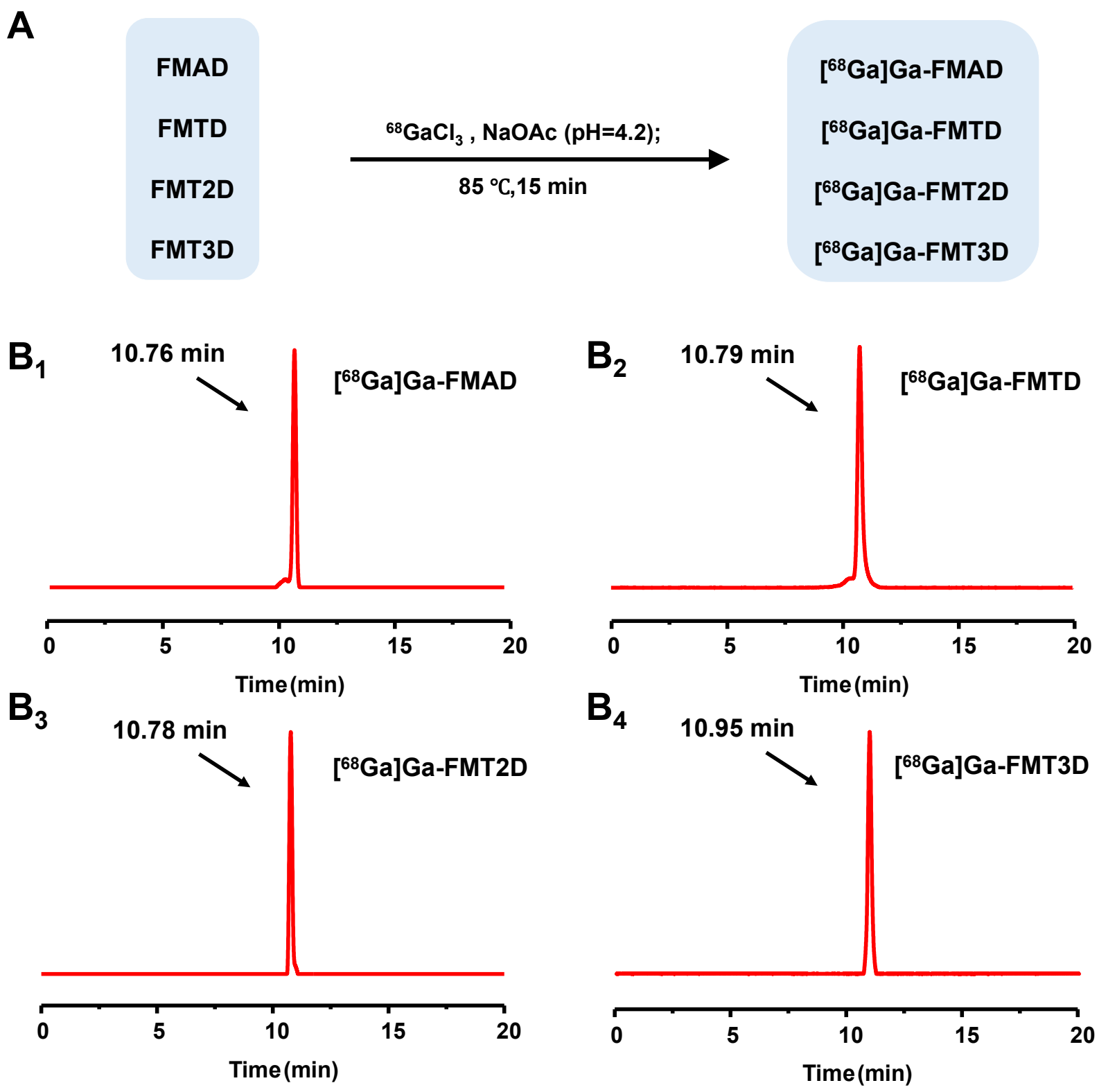

Figure S10 (A) Diagram illustrating radiolabeling. (B) HPLC analysis of the radiochemical purity of [^{68}Ga]Ga-FMAD, [^{68}Ga]Ga-FMTD, [^{68}Ga]Ga-FMT2D, and [^{68}Ga]Ga-FMT3D.

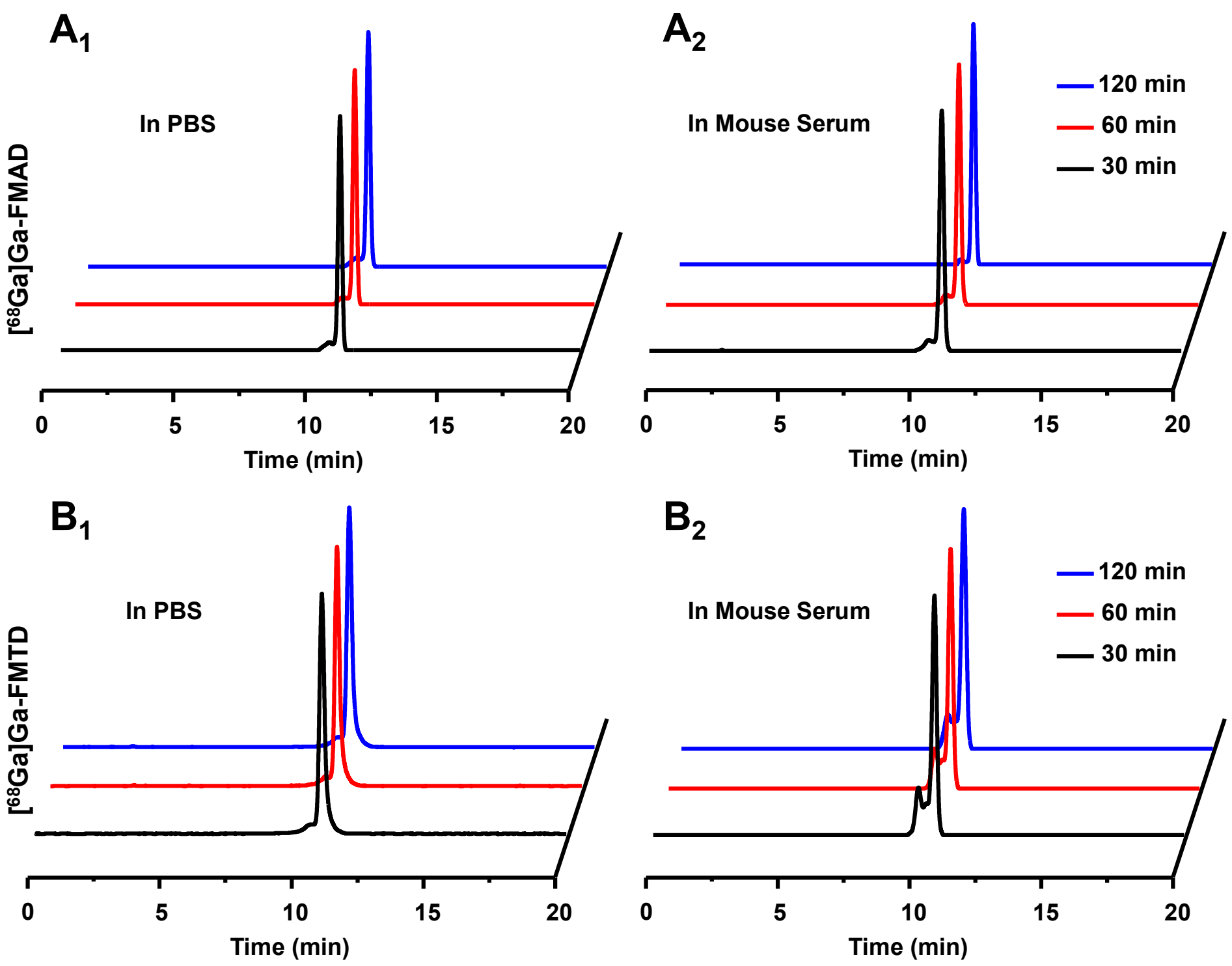

Figure S11 *In vitro* stabilities of [^{68}Ga]Ga-FMAD (A) and [^{68}Ga]Ga-FMTD (B) in PBS or mouse serum.

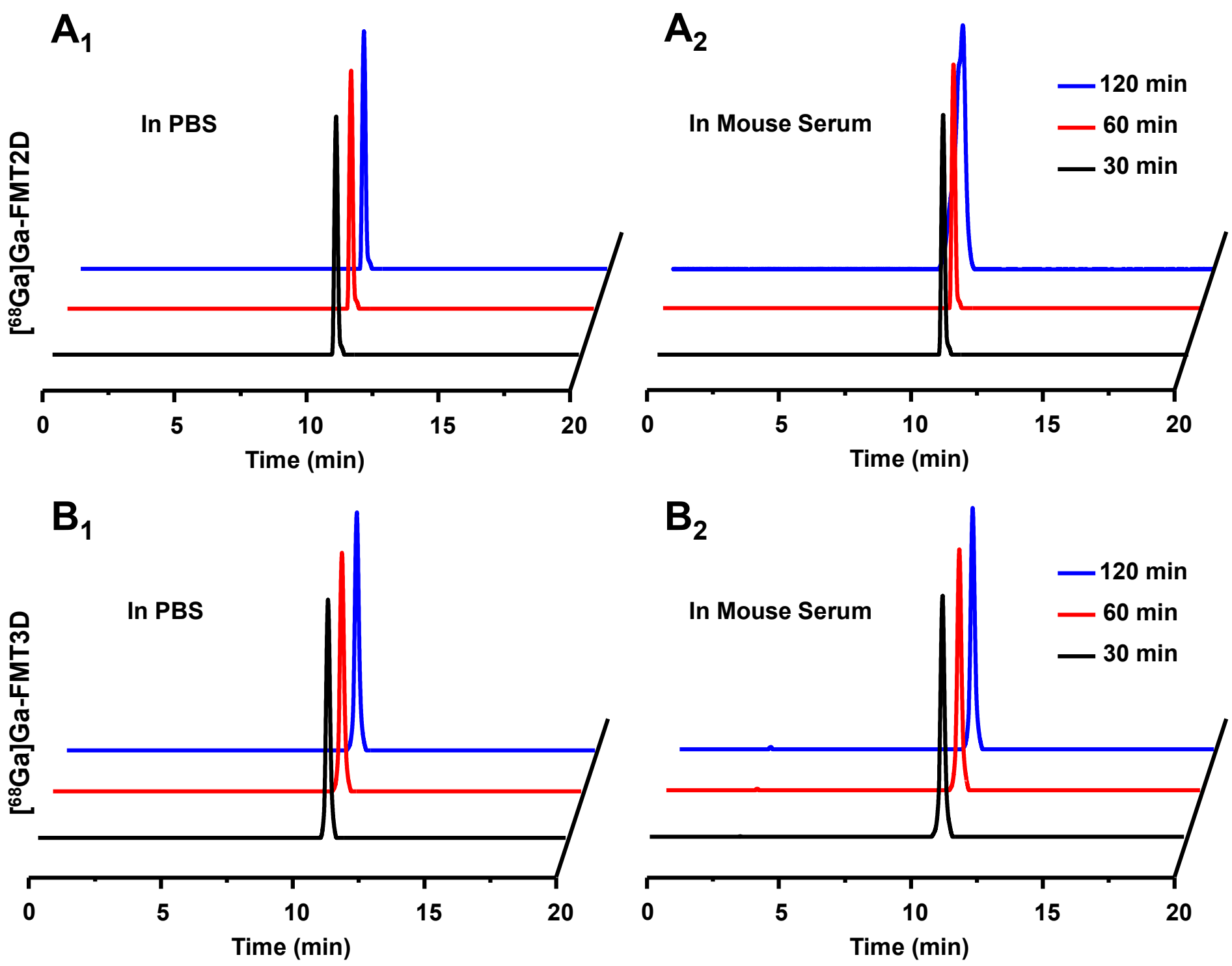

Figure S12 *In vitro* stabilities of [^{68}Ga]Ga-FMT2D (A) and [^{68}Ga]Ga-FMT3D (B) in PBS or mouse serum.

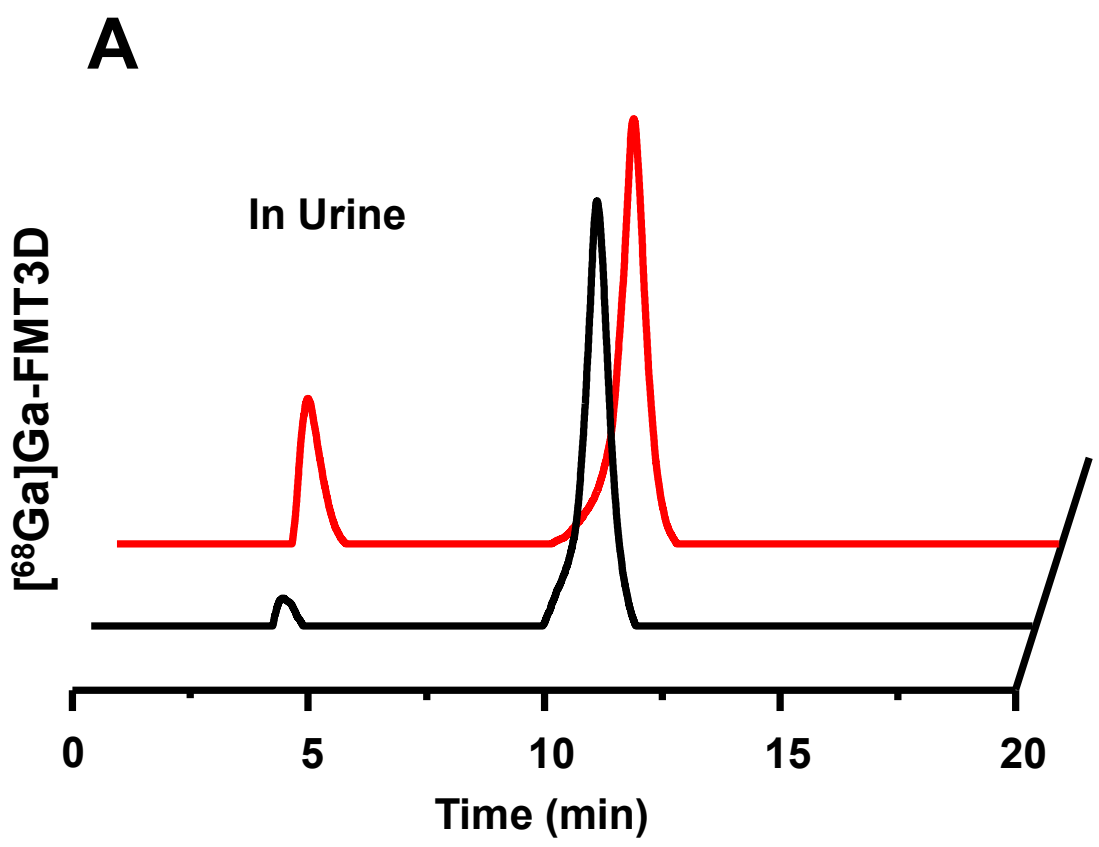

Figure S13 Metabolic stabilities of [^{68}Ga]Ga-FMT3D in the urine (A) or blood (B) of C57BL/6J male mice.

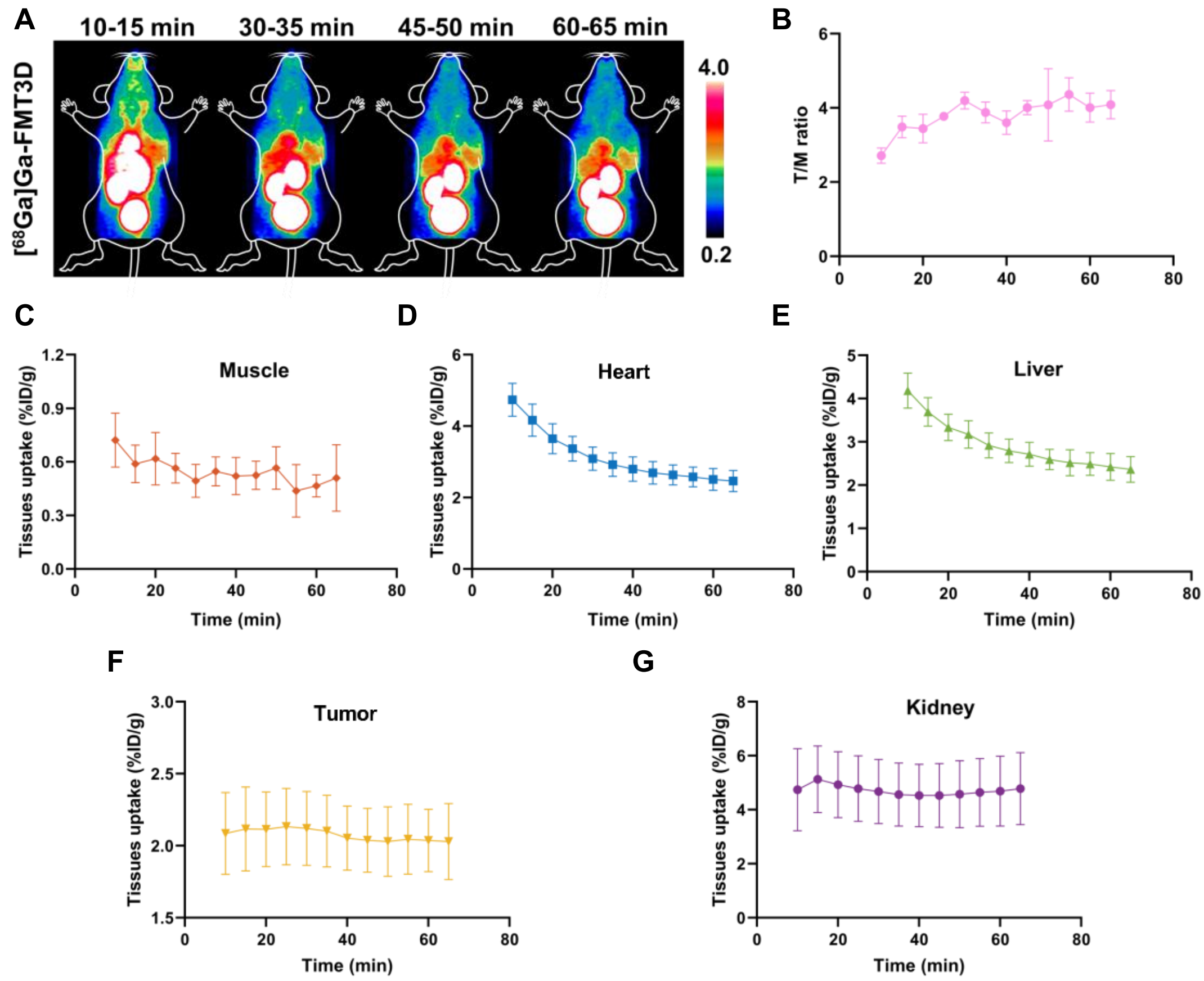

Figure S14 (A) Dynamic PET imaging of [^{68}Ga]Ga-FMT3D in B16F10 tumor-bearing mice. (B) The tumor-to-muscle ratio of [^{68}Ga]Ga-FMT3D were derived from PET images over time. The uptakes of muscle (C), heart (D, including contents), liver (E), tumor (F), and kidney (G) of [^{68}Ga]Ga-FMT3D were derived from PET images over time.

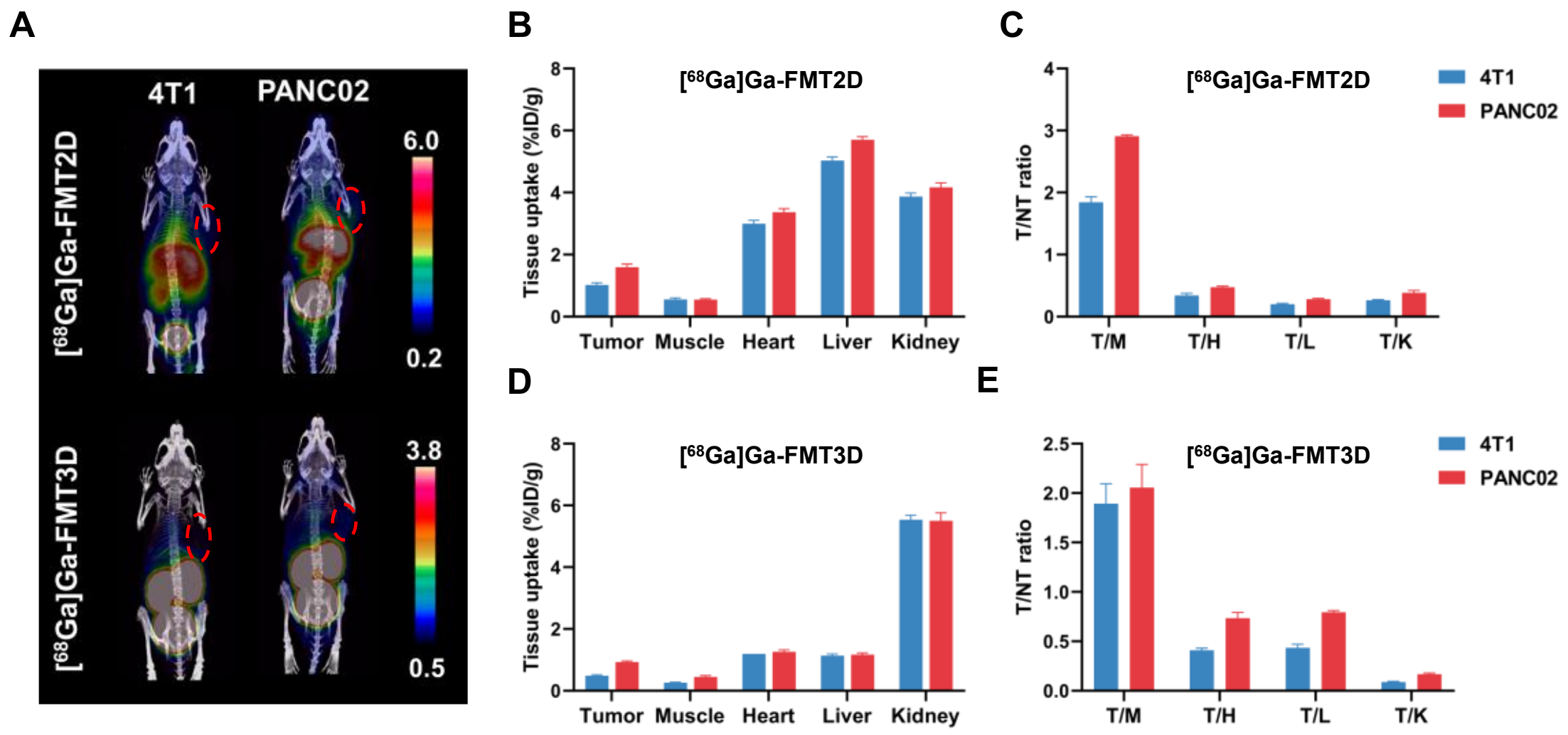

Figure S15 Small animal PET imaging of [^{68}Ga]Ga-FMT2D and [^{68}Ga]Ga-FMT3D at 1 h p.i. in 4T1 and PANC02 tumor-bearing mice. (A) Representative MIP images of radiotracers. The tumor uptake (B) and tumor-to-muscle ratio (C) of [^{68}Ga]Ga-FMT2D were derived from PET images. The tumor uptake (D) and tumor-to-muscle ratio (E) of [^{68}Ga]Ga-FMT3D were derived from PET images.

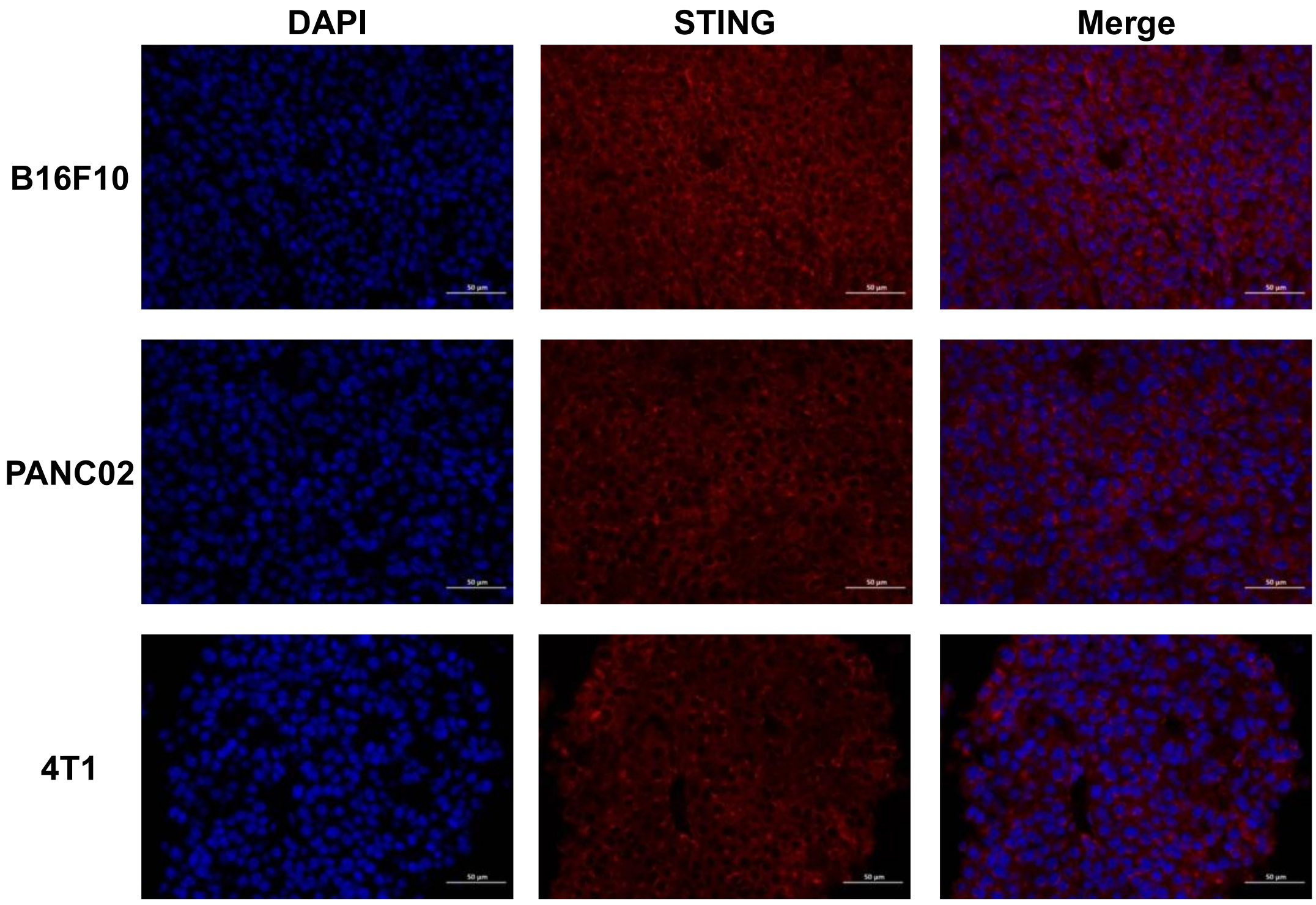

Figure S16 Representative immunofluorescence images of different tumor types.

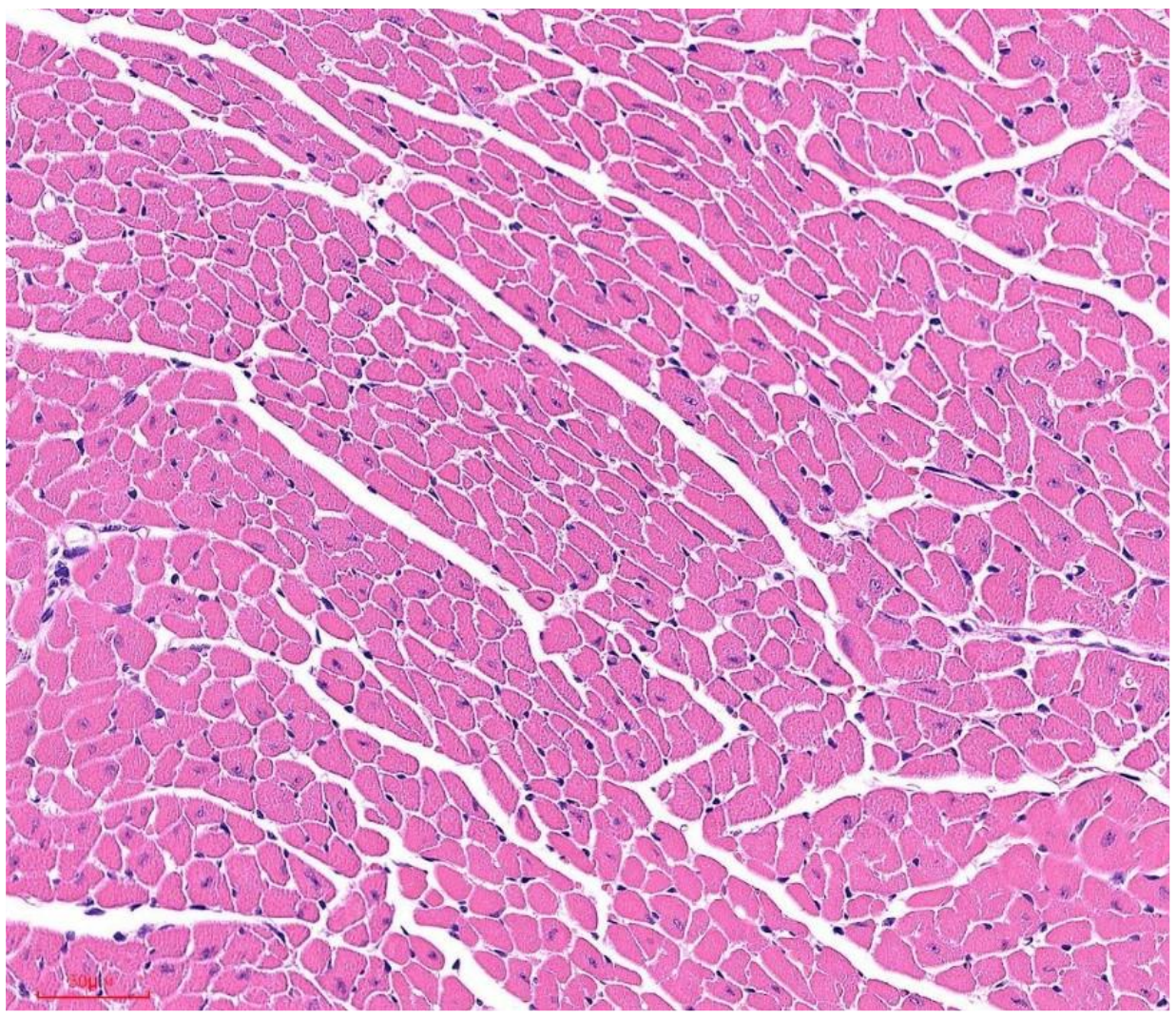

Figure S17 H&E staining of heart tissues after [^{68}Ga]Ga-FMT3D administration.

Table S1 Characteristics of [^{68}Ga]Ga-FMAD, [^{68}Ga]Ga-FMTD, [^{68}Ga]Ga-FMT2D, and [^{68}Ga]Ga-FMT3D.

Radioligand	RCY (%)	RCP (%)	A_m (GBq/μmol)	Log *P*
[^{68}Ga]Ga-FMAD	55.4 ± 7.0	> 97	7.0 ± 2.3	–1.55 ± 0.01
[^{68}Ga]Ga-FMTD	52.5 ± 6.4	> 97	7.5 ± 2.2	–2.12 ± 0.02
[^{68}Ga]Ga-FMT2D	63.0 ± 6.3	> 97	10.1 ± 7.1	–0.55 ± 0.01
[^{68}Ga]Ga-FMT3D	63.9 ± 7.8	> 97	13.9 ± 2.2	–1.77 ± 0.02

Table S2 Biodistribution results of [^{68}Ga]Ga-FMT2D and [^{68}Ga]Ga-FMT3D in B16F10 tumor-bearing mice at 1 h p.i.

Tissues	Uptake value (%ID/g)*	
	[^{68}Ga]Ga-FMT2D	[^{68}Ga]Ga-FMT3D
Heart	5.40 ± 0.95	2.73 ± 0.60
Liver	22.10 ± 6.98	4.66 ± 0.52
Lung	19.32 ± 4.37	7.17 ± 0.95
Kidney	17.13 ± 3.27	67.91 ± 6.64
Spleen	6.18 ± 1.99	1.86 ± 0.08
Stomach	4.14 ± 2.62	1.43 ± 0.11
Bone	2.98 ± 0.83	1.68 ± 0.23
Muscle	1.78 ± 0.53	0.97 ± 0.19
Intestines	10.96 ± 3.19	2.19 ± 0.16
Brain	0.56 ± 0.13	0.27 ± 0.14
Blood	19.46 ± 3.42	6.98 ± 0.94
Tumor	6.49 ± 0.79	5.56 ± 0.56

*Data presented as mean ± SD (n = 3).

Table S3 Tumor-to-normal tissue ratio of [^{68}Ga]Ga-FMT2D and [^{68}Ga]Ga-FMT3D in B16F10 tumor-bearing mice at 1 h p.i. derived from biodistribution results. T: tumor, H: heart (including contents), Li: liver, Lu: lung, K: kidney, Sp: spleen, St: stomach, B: bone, M: muscle, In: intestines, Br: brain, Bl: blood.

T/NT	[^{68}Ga]Ga-FMT2D	[^{68}Ga]Ga-FMT3D
T/H	1.33 ± 0.01	2.44 ± 0.73
T/Li	0.36 ± 0.02	1.28 ± 0.08
T/Lu	0.38 ± 0.03	0.87 ± 0.04
T/K	0.42 ± 0.03	0.08 ± 0.005
T/Sp	1.28 ± 0.18	3.06 ± 0.17
T/St	2.46 ± 0.07	4.13 ± 0.26
T/B	2.57 ± 0.15	3.65 ± 0.01
T/M	4.43 ± 0.81	6.12 ± 1.48
T/In	0.72 ± 0.15	2.40 ± 0.19
T/Br	13.28 ± 0.33	30.20 ± 2.70
T/Bl	0.37 ± 0.003	0.89 ± 0.08

Table S4 Estimated absorbed radiation dosimetry for [^{68}Ga]Ga-FMT3D in different organs (mSv/MBq).

Target organ	Mean	SD
Adrenals	6.03E-03	2.08E-03
Brain	1.30E-03	4.62E-04
Breasts	5.12E-03	1.78E-03
Gallbladder Wall	5.87E-03	2.01E-03
LLI Wall	1.75E-02	4.84E-03
Small Intestine	5.87E-03	2.03E-03
Stomach Wall	5.81E-03	2.01E-03
ULI Wall	6.26E-03	2.17E-03
Heart Wall	6.68E-03	2.34E-03
Kidneys	2.27E-02	8.45E-03
Liver	6.34E-03	1.59E-03
Lungs	1.49E-02	1.05E-02
Muscle	1.81E-03	6.32E-04
Ovaries	6.18E-03	2.13E-03
Pancreas	6.04E-03	2.08E-03
Red Marrow	4.91E-03	1.64E-03
Osteogenic Cells	8.82E-03	3.02E-03
Skin	4.72E-03	1.65E-03
Spleen	4.04E-03	1.39E-03
Thymus	5.55E-03	1.93E-03
Thyroid	5.26E-03	1.84E-03
Urinary Bladder Wall	5.28E-03	1.84E-03
Uterus	6.03E-03	2.10E-03
Total Body	5.81E-03	1.99E-03
Effective Dose Equivalent (mSv/MBq)	8.12E-03	2.58E-03
Effective Dose (mSv/MBq)	7.76E-03	2.37E-03
